# An EMT–Driven Alternative Splicing Program Occurs in Human Breast Cancer and Modulates Cellular Phenotype

**DOI:** 10.1371/journal.pgen.1002218

**Published:** 2011-08-18

**Authors:** Irina M. Shapiro, Albert W. Cheng, Nicholas C. Flytzanis, Michele Balsamo, John S. Condeelis, Maja H. Oktay, Christopher B. Burge, Frank B. Gertler

**Affiliations:** 1 Koch Institute for Integrative Cancer Research, Massachusetts Institute of Technology, Cambridge, Massachusetts, United States of America; 2 Computational and Systems Biology Program, Massachusetts Institute of Technology, Cambridge, Massachusetts, United States of America; 3 Department of Anatomy, Albert Einstein College of Medicine, Bronx, New York, United States of America; 4 Department of Pathology, Montefiore Medical Center, Bronx, New York, United States of America; 5 Department of Biology and Biological Engineering, Massachusetts Institute of Technology, Cambridge, Massachusetts, United States of America; University of Pennsylvania, United States of America

## Abstract

Epithelial-mesenchymal transition (EMT), a mechanism important for embryonic development, plays a critical role during malignant transformation. While much is known about transcriptional regulation of EMT, alternative splicing of several genes has also been correlated with EMT progression, but the extent of splicing changes and their contributions to the morphological conversion accompanying EMT have not been investigated comprehensively. Using an established cell culture model and RNA–Seq analyses, we determined an alternative splicing signature for EMT. Genes encoding key drivers of EMT–dependent changes in cell phenotype, such as actin cytoskeleton remodeling, regulation of cell–cell junction formation, and regulation of cell migration, were enriched among EMT–associated alternatively splicing events. Our analysis suggested that most EMT–associated alternative splicing events are regulated by one or more members of the RBFOX, MBNL, CELF, hnRNP, or ESRP classes of splicing factors. The EMT alternative splicing signature was confirmed in human breast cancer cell lines, which could be classified into basal and luminal subtypes based exclusively on their EMT–associated splicing pattern. Expression of EMT–associated alternative mRNA transcripts was also observed in primary breast cancer samples, indicating that EMT–dependent splicing changes occur commonly in human tumors. The functional significance of EMT–associated alternative splicing was tested by expression of the epithelial-specific splicing factor ESRP1 or by depletion of RBFOX2 in mesenchymal cells, both of which elicited significant changes in cell morphology and motility towards an epithelial phenotype, suggesting that splicing regulation alone can drive critical aspects of EMT–associated phenotypic changes. The molecular description obtained here may aid in the development of new diagnostic and prognostic markers for analysis of breast cancer progression.

## Author Summary

Epithelial-to-mesenchymal transition (EMT) is the process by which cancer cells lose their epithelial characteristics and obtain a mesenchymal phenotype that is thought to allow them to migrate away from the primary tumor. A better understanding of how EMT is controlled would be valuable in predicting the likelihood of metastasis and in designing targeted therapies to block metastatic progression. While there have been many studies on the contribution of changes in gene expression to EMT, much less is known regarding the role of alternative splicing of mRNA during EMT. Alternative splicing can produce different protein isoforms from the same gene that often have distinct activities and functions. Here, we used a recently developed method to characterize changes in alternative splicing during EMT and found that thousands of multi-exon genes underwent alternative splicing. Alternative isoform expression was confirmed in human breast cancer cell lines and in primary human breast cancer samples, indicating that EMT–dependent splicing changes occur commonly in human tumors. Since EMT is considered an early step in metastatic progression, novel markers of EMT that we identified in human breast cancer samples might become valuable prognostic and diagnostic tools if confirmed in a larger cohort of patients.

## Introduction

About 90% of human malignancies are carcinomas, tumors of epithelial origin [1]. The early steps in carcinoma metastasis often bear a striking resemblance to developmental programs involving Epithelial-to-Mesenchymal Transition (EMT), a process that converts organized epithelial cells into isolated, migratory cells with a mesenchymal morphology [2]. A growing body of work implicates EMT-like mechanisms in tumor cell invasion and dissemination in experimental systems and, recently, in human cancer [3], [4]. Normal epithelia are comprised of cells with aligned apical-basal polarity that are interconnected laterally by several types of junctions, including adherens junctions (AJs), which play important roles in establishing and regulating cell-cell adhesion [5]. During EMT, apico-basolateral polarity is lost, cell-cell junctions dissolve and the actin cytoskeleton is remodeled to endow cells with mesenchymal characteristics, including an elongated, migratory and invasive phenotype. Importantly, as a consequence of EMT cells may escape tumors, invade the surrounding tissue and migrate towards blood- or lymphatic vessels guided by the cells and extracellular matrix present in their microenvironment [6].

While EMT is thought to promote carcinoma invasion and metastasis, it is clear that other mechanisms for carcinoma progression exist [3], [7], and direct *in vivo* evidence linking EMT to metastasis in clinical subjects has been challenging to obtain. Some studies have shown that a poor clinical outcome correlates with markers of EMT progression [8]–[11]. Conversely, some reports have identified carcinoma cells in primary and metastatic lesions with well-differentiated epithelial morphology [7], [12]. Detection of EMT *in vivo* during metastasis is complicated further by a reverse process, Mesenchymal-to-Epithelial transition (MET), that is also important during embryonic development and is thought to occur during metastatic colonization at secondary sites [13]. New approaches are needed to detect EMT and MET during metastatic progression and to clarify their clinical significance [14], [15].

The molecular mechanisms underlying EMT have been studied extensively in the last decade. EMT-inducing growth factors can trigger signaling cascades that activate a network of transcription factors, including Snail, ZEB-1, Goosecoid, FOXC2, Twist and others [16], that orchestrate the EMT program. Ectopic expression of a number of the EMT-associated transcription factors can initiate the program as well. Twist, a potent EMT driver, was identified originally as an inducer of mesoderm formation in *Drosophila* [17]. Ectopic Twist expression in epithelial cells results in loss of E-cadherin-mediated cell-cell adhesion, acquisition of mesenchymal markers and increased motility of isolated cells [18], a hallmark of the mesenchymal phenotype.

EMT is also likely regulated by post-transcriptional mechanisms including alternative pre-mRNA splicing. Alternative splicing expands the diversity of the proteome by producing multiple mRNA and protein isoforms per gene [19]. More than 90% of human genes are estimated to undergo alternative splicing, with a majority of alternative splicing events exhibiting tissue-specific splicing differences [20]. A variety of cancer-associated genes express alternatively spliced isoforms [21], indicating that regulation at the level of splicing may play important roles in cancer onset and progression. Alternative splicing of FGFR2 correlates with EMT in rat bladder carcinoma cells, where mutually exclusive inclusion of one of two exons defines the ligand binding specificity of the receptor during EMT [22]. ENAH (also known as Mena), an actin cytoskeleton regulatory protein, contains a small coding exon 11a that is included only in epithelial cells and excluded in mesenchymal cell lines and during EMT [23], [24]. Alternative splicing of p120catenin (CTNND1) generates protein isoforms that display opposite effects on cell motility in epithelial and mesenchymal cells [25]. Recently, two epithelial-specific RNA binding proteins, ESRP1 and ESRP2, homologs of the nematode splicing factor Sym-2, were identified in a screen for regulators of FGFR2 splicing [24]. The RBFOX2 splicing factor has recently been demonstrated to regulate subtype-specific splicing in a panel of breast cancer cell lines [26]. The ESRPs and RBFOX2 promote epithelial splicing of a number of transcripts (including FGFR2 and ENAH), some of which play important roles in EMT [24], [27]. Loss of ESRPs in epithelial cells induces some EMT-like changes in cell morphology [28]. However, the full extent of alternative splicing during EMT and its functional consequences to cell phenotype has yet to be elucidated.

We used an established *in vitro* model of EMT to evaluate the amount of gene expression and alternative splicing changes during EMT. Using deep sequencing analysis of the transcriptomes of epithelial and mesenchymal cells, we discovered a global alternative splicing program that alters splicing of key regulators of cell phenotype, including proteins that control cell adhesion and cytoskeletal dynamics. Our analysis indicates that EMT-associated splicing is likely regulated by several splicing factors, including the ESRPs and members of the RBFOX, CELF, MBNL, and hnRNP classes of splicing factors. We found that partial induction of the epithelial splicing program in mesenchymal cells via ectopic expression of ESRP1 or by depletion of RBFOX2 conferred epithelial properties to mesenchymal cells, supporting a key role for alternative splicing during MET. Multiple EMT-associated alternative splicing events were identified in breast cancer cell lines and in primary human breast cancer samples where epithelial and mesenchymal splicing patterns were negatively correlated. This EMT-associated splicing signature likely represents a broadly conserved program involved in the acquisition of mesenchymal-like phenotypes *in vivo* that could be used to detect EMT in primary human cancers with a potentially significant prognostic value.

## Results

### Large-scale changes in gene expression accompany EMT

**Figure 1:**
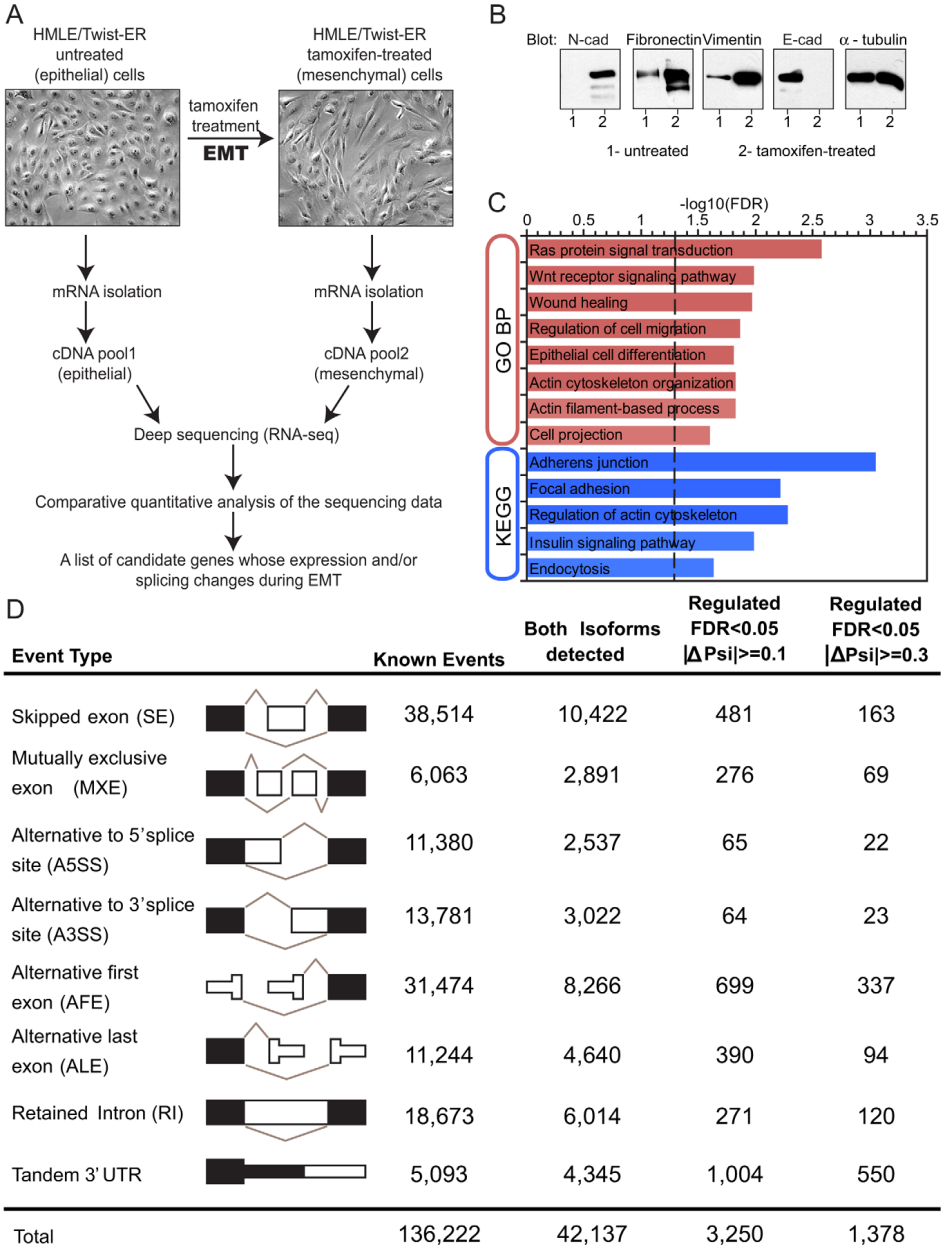
Alternative mRNA isoform expression in EMT. (A) Schematics of the *in vitro* EMT induction experiment. Immortalized human mammary epithelial cells (HMLE) expressing Twist fused to Estrogen Receptor (ER) were induced to undergo EMT by addition of tamoxifen into the culture media. mRNA was collected before EMT induction (epithelial sample) and after EMT induction (mesenchymal sample). cDNA pools from both samples were deep sequenced (RNA-Seq) and analyzed (See Materials and Methods). (B) Western blot analysis of N-cadherin, E-cadherin, fibronectin and vimentin expression with antibodies as indicated in cell lysates that were obtained before (1- untreated) and after (2- tamoxifen-treated) induction of EMT in HMLE/Twist-ER cells. α- tubulin was used as a loading control. (C) Gene ontology enrichment analysis bar graph of changes in alternative splicing events with |ΔΨ|> = 10% between samples. Gene ontology ‘biological process’, GO_BP_FAT, annotation is indicated in red on the y axis. KEGG Pathway (http://www.genome.jp/kegg/) annotation is indicated in blue on y axis. Benjamini FDR (−log10) is indicated on the x axis. Vertical dotted line marks Benjamini FDR = 0.05. (D) Column 1 shows different kinds of splicing events that have been analyzed. Columns 2–5 show the number of events of each type: (2) all known events based on AceviewAceView annotation; (3) events with both isoforms supported by RNA-Seq reads; (4) events detected at a False Discovery Rate (FDR) of 5% with ΔΨ > = 10% between samples; (5) events detected at an FDR of 5% with ΔΨ> = 30% between samples.

To assess gene and alternative mRNA isoform expression during EMT, we utilized an *in vitro* model in which mammary epithelial cells (HMLE) expressing Twist fused to a modified estrogen receptor (ER) undergo EMT when the fusion protein is activated by addition of the ER ligand 4-hydroxytamoxifen (4-OHT; tamoxifen) [29]. Untreated HMLE/Twist-ER epithelial cells maintained highly organized cell-cell adhesions and cell polarity (Figure 1A). Following tamoxifen treatment, the cobblestone-like appearance of HMLE/Twist-ER cells was replaced by a spindle-like, fibroblastic morphology, consistent with previously published results (Figure 1A; [29]). This morphological transformation represents one of the hallmarks of EMT. As expected, phenotypic changes coincided with a change in expression of canonical EMT markers, including loss of E-cadherin and induction of N-cadherin, Fibronectin and Vimentin expression (Figure 1B). Tamoxifen competes with estrogen for binding to ER to form a complex that translocates into the nucleus where it recruits co-repressors of transcription, thus preventing activation of ER downstream targets [30]. Since HMLE cells do not express endogenous ER (Figure S1B), EMT induction in HMLE/Twist-ER cells is likely initiated exclusively by downstream targets of Twist, making HMLE/Twist-ER cells a useful *in vitro* model of EMT.

To obtain an in-depth analysis of gene expression and splicing changes during EMT, we collected mRNA from untreated (epithelial) and from tamoxifen-treated (mesenchymal) HMLER/Twist-ER cells. Deep sequencing of fragments of polyA-selected mRNAs (RNA-Seq) was used to obtain a digital inventory of gene and mRNA isoform expression (Figure 1A). Between 27 million and 30 million 39-base-pair (bp) cDNA fragments were sequenced from each sample (Figure S1A). Sequenced cDNA fragments (reads) were mapped to the human genome (hg18 version) and to a splice junction database derived from AceView annotation [31]. In total, ∼75% of reads mapped uniquely to the genome or to splice junctions, allowing up to 2 mismatches. Less than 1% of total reads mapped uniquely to rRNA sequences (data not shown). Read density (coverage) was over 400-fold higher in exons than in introns or intergenic regions (Figure S1C), indicating that most reads derived from mature mRNA.

We first estimated gene expression changes during EMT using ‘Reads Per Kilobase of Exon Model per Million Mapped Reads’ (RPKM), a measure of expression that reflects the molar concentration of a transcript in the sample by normalizing read counts for mRNA length and for the total read number in the sample [32]. Applying both a statistical cut-off based on Audic-Claverie statistics for read-based expression profiling [33] and requiring a minimum 3- fold change, we observed that ∼2,060 genes were downregulated, while ∼950 were upregulated in EMT (Figure S2A), indicating a large-scale reorganization of the transcriptome during this process in agreement with recently published data [34]. As expected, E-cadherin was downregulated, while N-cadherin was upregulated during EMT [18]; actin transcript levels remained unchanged (Figure S2A). These observations revealed that Twist-induced EMT is accompanied by massive changes in gene expression similar to those observed in developmental EMT [35].

Gene ontology (GO) enrichment analysis of up- and down-regulated genes was used to gain insight into functional significance of the EMT-driven expression changes. Genes involved in epithelial cell differentiation, encoding components of cell cycle machinery and cell-cell junction components, were downregulated during EMT (Figure S2B). Concomitantly, genes associated with cell-matrix adhesion, extracellular matrix organization and cell motility, were upregulated (Figure S2C). Thus, the most significant EMT-driven changes in gene expression are associated with gene categories involved in the phenotypic conversion that occurs during EMT, in agreement with previously published data [36].

### Alternative isoform expression is grossly affected in EMT

To explore the extent of regulated RNA processing during EMT, we examined eight common types of alternative isoform expression events, each capable of producing multiple mRNA isoforms from a gene through alternative splicing, alternative cleavage and polyadenylation (APA) and/or alternative promoter usage (Figure 1D). These eight types of events included: skipped exons (SE), retained introns (RI), mutually exclusive exons (MXEs), alternative 5′ and 3′ splice sites (A5SS and A3SS), alternative first exons (AFE), alternative last exons (ALE) and tandem 3′ untranslated regions (tandem 3′ UTRs). A comprehensive set of ∼136,000 events of these eight types was derived from the AceView gene annotations [31]. The fraction of mRNAs that contained an alternative exon – the ‘percent spliced in’ (PSI or Ψ) value – was estimated by the ratio of the density of inclusion reads to the sum of the densities of inclusion reads and exclusion reads, with a variant of this method used for tandem 3′ UTRs, as described previously [20]. Thus, Ψ values range from ∼0, indicating predominant exclusion of an alternative exon from mRNAs, to ∼1, indicating predominant inclusion of the exon.

The extent of EMT-specific regulation of these events was assessed by comparison of the mesenchymal (post-EMT) to the epithelial (pre-EMT) RNA-Seq data (Figure 1D). In all, for ∼40% of genes with documented alternative isoforms, both isoforms were detected by RNA-Seq reads. Of the events where both isoforms were detected, about 1 in 10 skipped exons and 1 in 20 mutually exclusive exons exhibited a significant change in Ψ value >10%, with hundreds of alternative splicing events of other types also regulated at this level (Figure 1D). At the gene level, 4.5% of genes contained an event(s) with an absolute change in Ψ value greater than 10% during EMT, and 2% of genes contained an event(s) with a Ψ value change greater than 30% (Table S1). These data indicate that a substantial change in splicing accompanies EMT.

To confirm the accuracy of RNA-Seq analysis of alternative splicing during EMT, a subset of SE and MXE events was chosen from the set with False Discovery Rate (FDR) below <0.05 and |ΔΨ|>0.1 for semi-quantitative RT-PCR (sqRT-PCR) analysis using cDNA from cells before and after EMT induction. Alternative splicing events with |ΔΨ|>0.1 between human tissues are enriched for evolutionarily conserved sequences surrounding the alternative exons as compared to constitutive exons, suggesting that use of this cutoff enriches for functional events [20]. The tested subset included 37 alternative exons that showed relatively large changes in splicing based on the analysis of the RNA-seq data, or whose host genes encoded functionally interesting molecules with respect to EMT (e.g., adhesion molecules). This subset also included a few events that showed relatively small changes in isoform expression in order to assess the robustness of our statistical test. In all cases, the change in splicing ΔΨ ( = Ψ_M_−Ψ_E_) detected by RT-PCR was in the same direction as that determined by RNA-Seq (Figure S1D), and in 78% of cases, the change in Ψ observed by sqRT-PCR was 20% or higher. Altogether, a strong concordance (R^2^ = 0.86; Figure S1D) was observed between splicing changes detected by RNA-Seq and measurements by sqRT-PCR. The high validation rate and quantitative concordance by an independent method (sqRT-PCR) support the reliability of the alternative splicing events identified by the RNA-seq analysis.

Genes with altered splicing during EMT showed strong enrichment for involvement in biological processes related to the regulation of the actin cytoskeleton, cell-cell junctions, regulation of cell migration and wound healing. Pathway analysis using KEGG and GO detected enrichment of EMT-associated alternative splicing events in the Wnt, Ras and Insulin pathways (Figure 1C). These enriched terms suggested that alternative splicing plays a role in pathways that direct morphological and motility-related changes associated with EMT. Interestingly, although the types of gene functions most affected at the splicing and expression levels were largely similar, the actual sets of genes undergoing splicing-level and expression-level changes did not overlap more than expected by chance (Figure S3). This observation suggests that the EMT splicing program functions in a manner that is parallel to the transcriptional program and that gene expression and alternative splicing, may coordinately drive changes to specific aspects of cell morphology related to the cytoskeleton, cell adhesion and cell motility.

### Regulatory motifs and factors associated with the EMT splicing program

**Figure 2:**
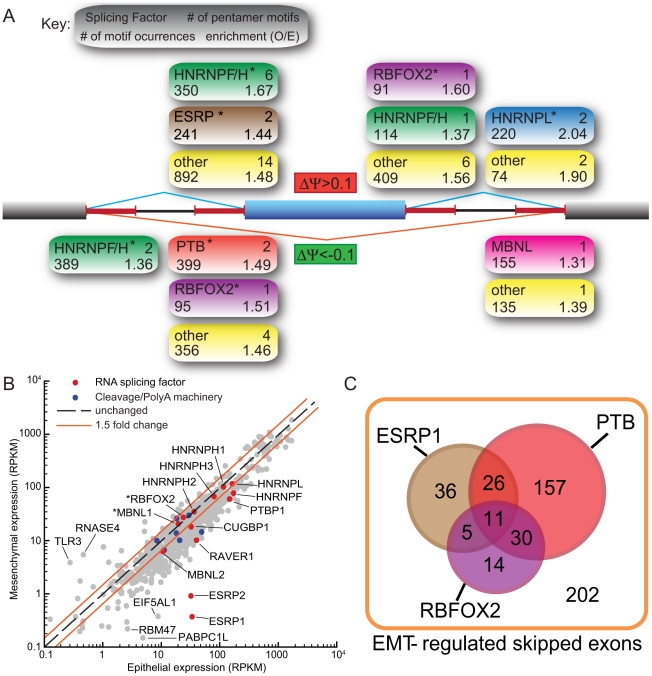
Motif analysis reveals splicing factors that are involved in the regulation of EMT–specific splicing. (A) Pentamer motifs significantly enriched (FDR<0.1) in the 4 flanking 250-nt intronic regions of EMT-regulated skipped exons. Statistics of motifs resembling known binding sites of splicing factors are annotated as described in the key. Motifs that are not recognized as known binding sites are grouped into the “Other” group. * = at least one known motif of that splicing factor has an FDR<0.05. (B) Scatter plot of expression levels of RNA binding proteins and mRNA splicing regulators in epithelial and mesenchymal cells. Some splicing factors whose motifs were enriched in (A) are highlighted. Asterisks mark splicing factors which are also regulated by alternative splicing of their mRNA transcripts. Genes encoding components of cleavage/polyadenylation machinery are also highlighted. (C) A venn diagram showing potential regulation of EMT-associated skipped exon events by ESRP1, PTP and RBFOX2 splicing factors based on the microarray analysis of ESRP1 depleted MDA-MB-231 cells [28] and CLIP-Seq analysis of FOX and PTB [27], [37] (See Text S1). The universe of the Venn diagram consists of all EMT-regulated SE events by FDR of 5% and |ΔΨ|> = 10%. P (FOX2) = 8.58e-05; p (PTB) = 0.0013; p (ESRP1) = 9.27e-16.

A substantial shift in the levels or activity of the major splicing factors likely underlies the large-scale program of splicing changes that occur during EMT. To explore the nature of this shift, we first analyzed the incidence of oligonucleotide motifs occurring in regulated alternative transcripts. As most splicing factors bind short RNA oligomers a few bases long, we identified pentanucleotides (5mers) that were enriched in regions adjacent to the splice sites involved in the splicing of exons induced or repressed upon EMT (Figure 2A). This analysis identified a few dozen 5mers enriched in each region relative to control alternative introns, including motifs corresponding to the RBFOX, CELF, ESRP and MBNL families of tissue-specific factors, as well as motifs for several heterogeneous nuclear ribonucleoprotein (hnRNP) factors, including hnRNPs F and H, PTB/hnRNP I, and hnRNP L (Table S2, S3). A subset of these motifs was specifically enriched adjacent to exons whose Ψ values increased following EMT relative to exons whose splicing did not change (Figure 2A). These included motifs associated with RBFOX and ESRP splicing factors and with hnRNPs F/H and L. An overlapping subset of motifs were enriched adjacent to exons whose Ψ values decreased following EMT, again including motifs associated with the RBFOX and hnRNP F/H families and also motifs associated with PTB and MBNL family proteins (Figure 2A). Several 5-mers, without clear RNA binding protein partners, that may represent binding sites of uncharacterized splicing regulators in EMT were also identified.

We also examined changes in the expression of RNA binding protein (RBP) genes. The most striking changes in RBP expression occurred for the related epithelial specific splicing factors ESRP1 (RBM35A) and ESRP2 (RBM35B) [24]. During EMT, the expression of these factors decreased by ∼90-fold and ∼35-fold, respectively, from relatively high initial levels (Figure 2B). Motif enrichment for ESRP splicing factors was observed in the upstream sequence of cassette exons upregulated during EMT (Figure 2A) consistent with the recent observation that ESRP binding sites are present at greater numbers upstream of silenced exons, than included exons [28]. As ESRPs are downregulated during EMT, these silenced exons are relieved from ESRP inhibition and thus appear upregulated.

Splicing factor activity often switches between positive and negative regulation depending on the location of binding relative to the regulated exon. RBFOX family splicing factors tend to enhance splicing when bound downstream and to repress splicing when bound upstream of alternative exons [27]. The observed pattern of enrichment of RBFOX motifs downstream of exons whose inclusion increased during EMT and upstream of exons whose inclusion decreased (Figure 2A) is therefore consistent with an increase in the activity of RBFOX family factors during EMT. Expression of the RBFOX2 gene increased moderately but significantly by about 15% following EMT (Table S4) while at the same time splicing of a MXE encoding the RNA-binding domain of the RBFOX2 protein increased by about 20%. Thus, these changes together should increase the levels of splicing-active RBFOX2 mRNA by at least a third. Recently, it has been suggested that RBFOX2 activity plays a role in regulating a set of breast cancer subtype–specific alternative splicing events [26]. The expression levels of many other RBPs associated with motifs enriched near EMT-regulated exons changed during EMT (Figure 2B, Table S4), including downregulation of the splicing repressor PTBP1 (PTB/hnRNP I) by ∼2.5-fold, downregulation of the PTB-associated splicing co-repressor RAVER1 by ∼4-fold, and downregulation of the myotonic dystrophy-associated splicing factors MBNL2 and MBNL3 and hnRNP F by ∼1.6- to 2.5-fold. These observations suggested that changes in the levels and activity of several different splicing factors may contribute to the splicing changes observed in EMT.

**Figure 3:**
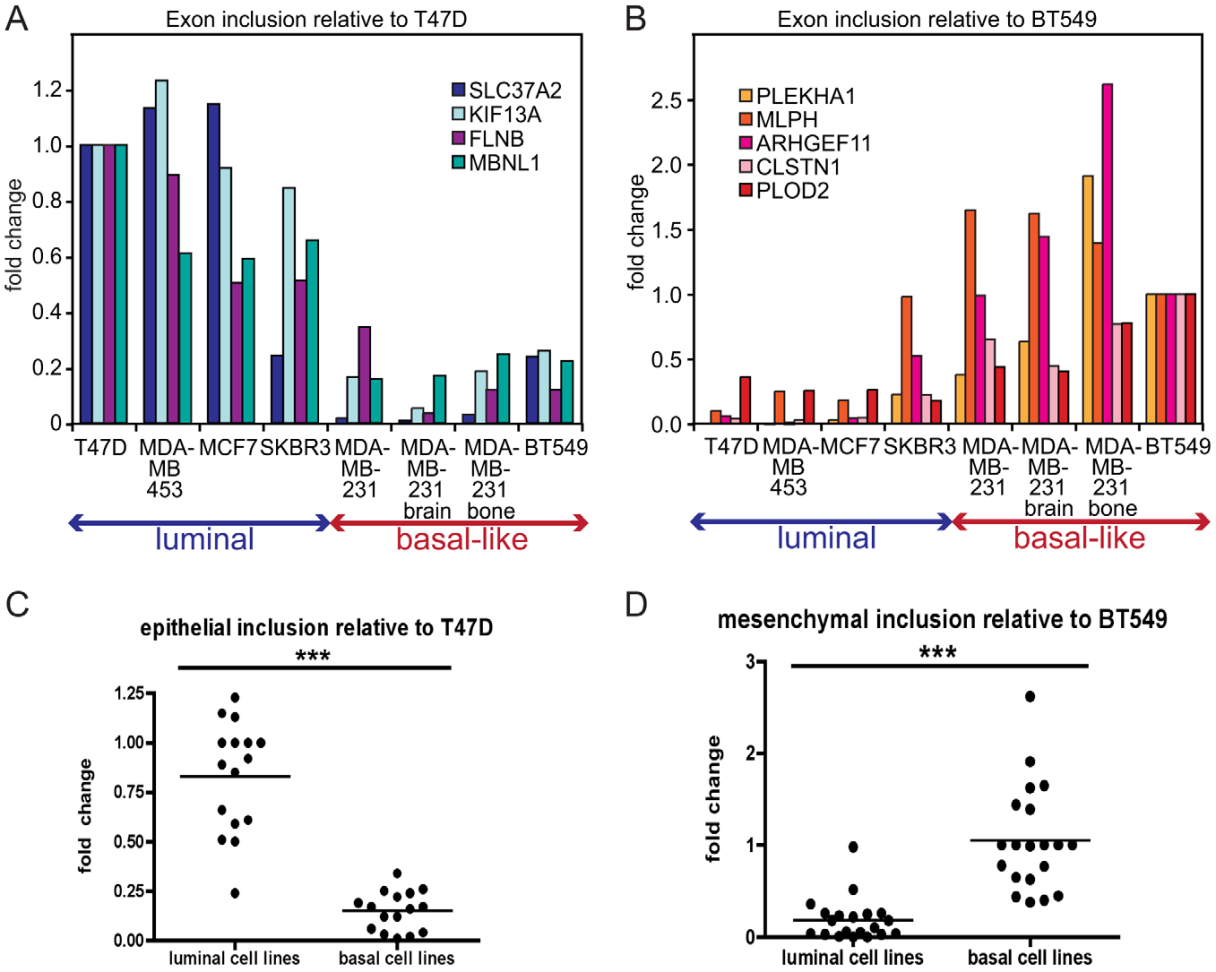
EMT–associated alternative splicing events are confirmed in breast cancer cell lines. (A) Alternative exon inclusion in four mRNA transcripts, as indicated, in eight breast cancer cell lines determined by a qRT-PCR analysis and depicted as a fold change relative to exon inclusion in T47D luminal cell line. (B) Alternative exon inclusion in five mRNA transcripts, as indicated, in eight breast cancer cell lines depicted as a fold change relative to exon inclusion in BT549 basal B cell line. (C) Distribution of all epithelial inclusion events combined. Each event is depicted as a fold change relative to inclusion in T47D. (D) Distribution of all mesenchymal inclusion events combined. Each event is depicted as a fold change relative to inclusion in BT549 cells. For (C) and (D), *** = p<0.001.

To explore the potential contributions of splicing factors to EMT-regulated alternative splicing, we analyzed published cross-linking/immunoprecipitation-sequencing (CLIP-Seq) data from human cell lines. Dozens of EMT-regulated skipped exons were associated with RBFOX2 CLIP-Seq clusters, and hundreds were associated with PTB CLIP-Seq clusters (Figure 2C; [27], [37]). In addition, a fraction of the observed EMT-regulated splicing events overlapped with a set of ESRP1-regulated exons recently identified by Carstens and coworkers using RNAi and a splicing-sensitive microarray analysis (Figure 3C; [28]). Together, the RNAi and CLIP-Seq data demonstrate the potential for regulation of a substantial portion – perhaps a majority of EMT-regulated exons – by these three factors. Thus, our data are consistent with a model in which several splicing factors collaborate in the regulation of splicing during EMT, adding a layer of post-transcriptional regulation to the EMT program.

### EMT–associated alternative transcripts correlate with the phenotype of breast cancer cell lines

Alternatively spliced mRNA isoforms that exhibit EMT-associated changes in exon inclusion might serve as valuable prognostic markers for metastatic disease, since EMT is considered an early event in metastatic progression. As an initial step towards eventual analysis of primary human samples, we assessed alternative isoform expression in a panel of human breast cancer cell lines of luminal (generally poorly metastatic) and basal-like origin (generally aggressive and metastatic). Luminal cell lines, like MCF7 and T47D, express high levels of epithelial markers including E-cadherin, while basal-like cell lines express mesenchymal markers including N-cadherin, vimentin and fibronectin [14]. In addition, in our analysis we included two cell lines – derivatives of MDA-MB-231 cell metastases to the brain and bone – that exhibited a more aggressive phenotype compared to the parental MDA-MB-231 cells [38]. We hypothesized that splicing events with high epithelial inclusion (i.e. high inclusion in the pre-EMT/epithelial sample) would be expressed in luminal breast cancer cell lines, and conversely that splicing events with high mesenchymal inclusion (defined analogously) would be expressed in basal-like cell lines. A quantitative RT-PCR (qRT-PCR) analysis of nine skipped exons demonstrating the largest ΔΨ in the validated set of 37 alternative splicing events, using cDNA from the panel of luminal and basal-like cell lines, indicated that four epithelial inclusion events, in the SLC37A2, KIF13A, FLNB, and MBNL1 genes, were included at high frequency in luminal cell lines, whereas inclusion of these events was low in basal-like cells compared to T47D epithelial cells (Figure 3A). Conversely, five mesenchymal-enriched inclusion events in the PLEKHA1, MLPH, ARHGEF11, CLSTN1 and PLOD2 genes were enriched in basal-like cell lines with only low inclusion levels in luminal cells relative to BT549 mesenchymal cells (Figure 3B), consistent with recently published results [28]. Thus, taken together, epithelial inclusion events were identified in corresponding mRNA transcripts in luminal cells and were detected at very low levels in basal-like cells, while mesenchymal inclusion events were detected at low levels in luminal cells but showed a high inclusion ratio in basal-like cells (Figure 3C, 3D). Therefore, the qRT-PCR analysis of skipped exons using cDNA from a panel of luminal and basal-like breast cancer cell lines detected EMT-associated splicing events, as predicted by the RNA-seq analysis of Twist-induced EMT.

**Figure 4:**
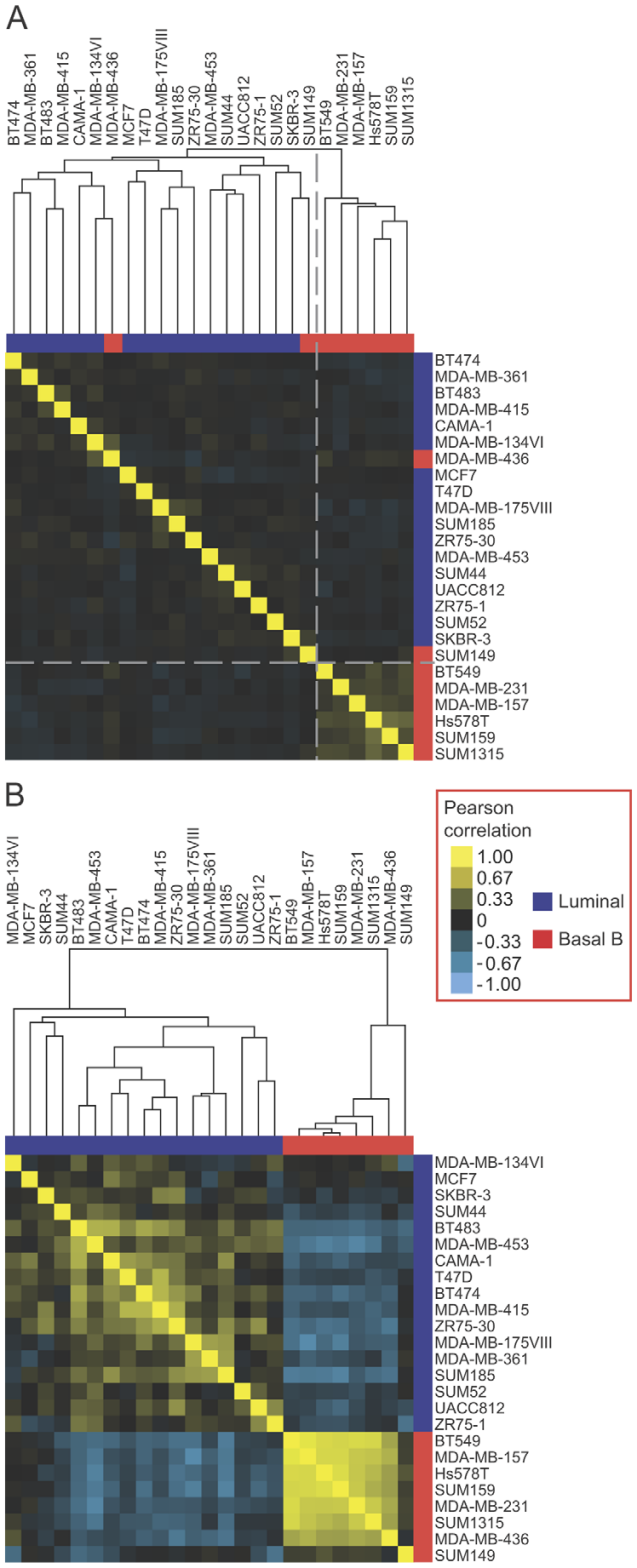
EMT–associated alternative mRNA isoforms classify breast cancer subtypes. (A) Unsupervised hierarchical clustering of NCI-60 breast cancer cell lines [39], as indicated, using the 307 EMT-associated SE events (|ΔΨ|>0.1, FDR<0.05). Luminal cell lines are marked in blue. Basal B cell lines are marked in red. Shades of yellow indicate positive pearson correlation. Shades of blue indicate negative pearson correlation. The color scale is shown on the right. The dotted line separates luminal and basal cell line clusters. (B) Clustering correlation matrix of NCI-60 breast cancer cell lines [39], as indicated, using the 24 coherent events.

To explore the expression of EMT-associated alternative splicing events in breast cancer cell lines further and to determine whether EMT-associated alternative exons could classify breast cancer cell line subtypes, we compared the expression of skipped exons (SEs) from our EMT RNA-seq analysis to available exon array data from luminal and basal B breast cancer cell lines in the NCI-60 panel [39]. Unsupervised hierarchical clustering of exon array data relating to 307 EMT-associated SE events (|ΔΨ|>0.1, FDR<0.05; foreground set) detected by the array, segregated basal B cell lines from luminal cell lines with only two basal cell lines (MDA-MB-436 and SUM149) misclassified in the luminal cluster (Figure 4A). In contrast, clustering of the exon array data using the background set of 8839 events resulted in cell line subtype classification with nine misclassifications, indicating lack of intrinsic bias in the whole set of analyzed events and that the SE events identified by our EMT RNA-Seq better classify the luminal and basal B cell lines. Furthermore, a randomized-clustering procedure demonstrated that the clustering classification using our set of SE events was statistically significant (p-value = 0.0014). Therefore, the EMT-associated splicing program identified by our RNA-seq analysis is conserved in breast cancer cell lines and correlates with their invasive and metastatic properties.

We hypothesized that some of the heterogeneity in splicing observed across the cell lines stemmed from cell type specific splicing events that may not be linked directly to EMT regulation (Figure 4A). To find the “core” EMT alternative splicing signature that can unambiguously distinguish between breast cancer cell line subtypes, we compared EMT-driven SE events to the SE events that were differentially regulated between the luminal and basal B cell lines. Of the SE events represented on the array that changed significantly in our EMT RNA-Seq dataset (|ΔΨ|>0.1, FDR<0.05), a total of 24 events changed significantly between luminal and basal B cell lines at an FDR<0.25. Of these, 19 (79%) changed in a “coherent” manner in the sense that the change in exon inclusion was in the same direction between mesenchymal and epithelial samples in the EMT RNA-seq dataset as between basal B and luminal cell lines in the exon array dataset (Figure S4). Interestingly, coherence increased for events that changed more dramatically in the EMT RNA-Seq dataset, with 11 (100%) of SE events (RNA-seq |ΔΨ|>0.3) exhibiting coherence between the two datasets (Figure S4). Notably, clustering analysis of luminal and basal B breast cancer cell lines using 19 coherent SE events demonstrated that luminal cell lines could be unambiguously distinguished from basal B cell lines based exclusively on these splicing events alone (Figure 4B). These “core” EMT-associated alternative splicing events may comprise a common program that contributes to the phenotypic changes that endow cancer cells with invasive and metastatic capabilities.

### Alternative isoforms detected in the *in vitro* EMT model are expressed in primary human breast cancer samples

**Figure 5:**
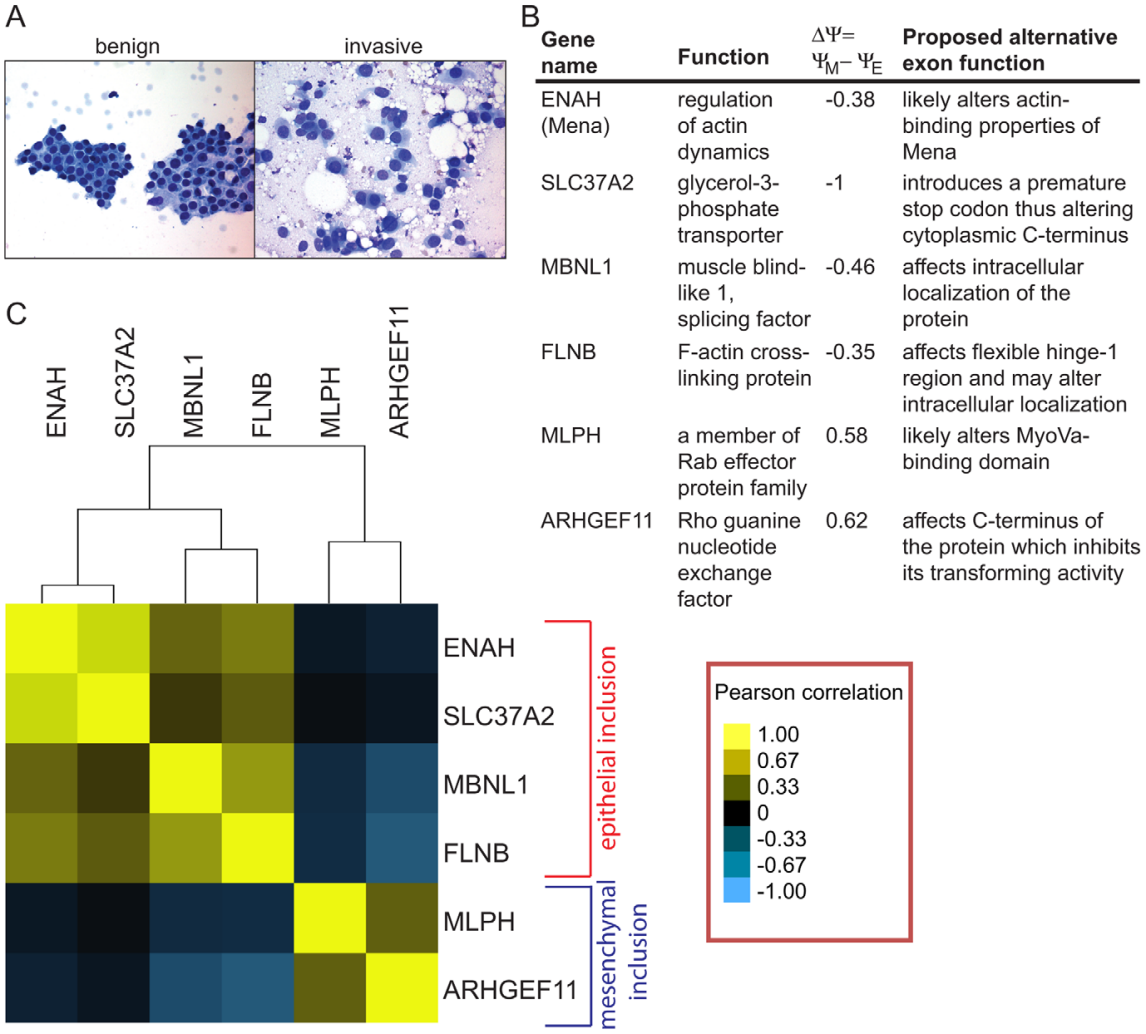
Alternative mRNA isoforms are expressed in FNA samples from breast cancer patients. (A) An example of a fine needle aspiration (FNA) spread from a benign and an invasive human breast tumor. (B) A table describing gene names, gene functions, change in inclusion levels during EMT (ΔΨ) and proposed functions of six SE events used in the FNA qRT-PCR analysis in (C). (C) Heat plot of pairwise Pearson correlation coefficients obtained from correlation analysis of exon inclusion ratios in 40 IDC samples normalized to an average fibroadenoma sample exon inclusion for six alternative splicing events. Yellow indicates a correlation of 1, black indicates a correlation of 0, light blue indicates a correlation of −1. Shades of yellow and blue mark correlation in-between.

To determine whether the alternative mRNA isoforms confirmed in human breast cancer cell lines are relevant to human disease, we assessed expression of these events in fine needle aspiration (FNA) biopsies from breast cancer patients. FNA is the least invasive available method of collecting diagnostic material from patients with breast mass. This procedure is performed using a small gauge needle that gently disrupts the tissue and allows loose tumor cells to travel up the needle via capillary action. The FNA sample is usually enriched in tumor cells and can be analyzed by qRT-PCR [40]. However, due to the small volume of the sample, RNA recovery is low – tens of nanograms of total RNA at most. As expected, a subset of patient FNA spreads contained tumor cells that appeared cohesive and tightly attached to each other, typical of benign ductal lesions, while another subset of FNA smears from invasive ductal carcinomas (IDCs) contained discohesive populations of enlarged tumor cells (Figure 5A), typical for a highly invasive phenotype. Analysis of 15 random FNA smears from IDCs used in this study for the percentage of tumor, inflammatory and stromal cells demonstrated an almost a complete absence of adipocytes, macrophages and inflammatory cells (Figure S5), indicating that all of the cells present in FNA samples were ductal cancer cells. Therefore, the phenotypic characteristics of FNA collected samples indicated that they represent an appropriate human sample for assessment of alternative mRNA transcript expression found in our *in vitro* screen for EMT-associated splicing.

To check expression of alternative mRNA isoforms, we obtained FNA samples from 40 patients with IDCs of various grades and growth hormone receptor status. IDCs in patients were classified as well, moderately or poorly differentiated according to the modified Bloom Richardson scale. The clinical and demographic data including patients' age, tumor size, lymph node status, estrogen, progesterone and Her2/neu receptor status were also collected (Table S5). Using the cDNA from 40 IDC samples, we determined inclusion ratios for six SE events that exhibited the largest change in exon inclusion levels based on the analysis of breast cancer cell lines. These included epithelial inclusion events in ENAH, MBNL1, FLNB and SLC37A2, and mesenchymal inclusion events in MLPH and ARHGEF11 (Figure 5B). The small amount of RNA isolated from FNA samples permitted analysis of only six alternative splicing events per sample. Inclusion ratios of splicing events in IDC samples were normalized to the average inclusion ratio of the same splicing event measured in six fibroadenoma (FA) samples. For each pair of splicing events, the Pearson correlation between normalized inclusion ratios of the two splicing events across 40 IDC samples was calculated and used for clustering analysis to assess the relationships between events (Figure 5C). Interestingly, ENAH and SLC37A2 as well as MLPH and ARHGEF11 inclusion events were highly correlated. Some epithelial and mesenchymal inclusion events were inversely correlated, e.g., increases in FLNB inclusion tended to be associated with decreases in inclusion of the ARHGEF11 alternative exon. Little or no correlation was observed between SLC37A2 and MLPH inclusion events. Overall, many IDCs expressed the mesenchymal mRNA isoforms, indicating that EMT-associated splicing occurs in human tumors *in vivo*.

Unsupervised clustering of splicing ratios of six alternative exons in 34 FNA samples demonstrated a significant correlation between the two mesenchymal markers, MLPH and ARHGEF11, and between the four epithelial markers, ENAH, SLC37A2, FLNB and MBNL1, while epithelial and mesenchymal marker groups exhibited anti-correlation (Figure S6A). Approximately Unbiased (AU) p-values obtained from the Pvclust analysis (http://www.is.titech.ac.jp/~shimo/prog/pvclust/) were >99%, thus supporting reliability of the clustering tree (Figure S6B). This result suggests that the IDC samples tended to have either epithelial or mesenchymal splicing patterns but rarely exhibited mixed inclusion patterns, indicating that IDCs could be unambiguously classified into two groups on this basis.

### The ESRP1 splicing factor confers epithelial-like properties to mesenchymal cells

**Figure 6:**
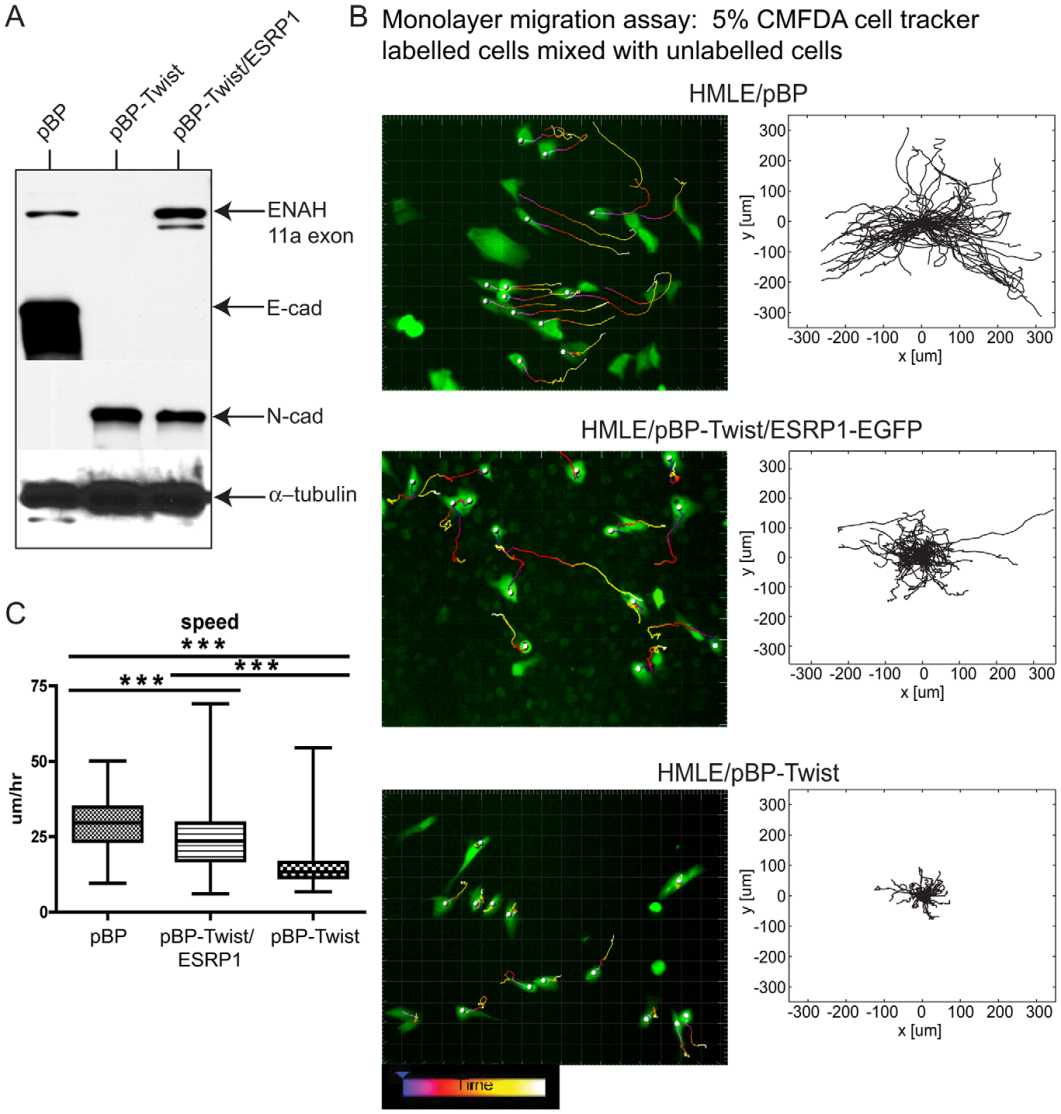
Expression of ESRP1 confers epithelial migration properties to mesenchymal cells. (A) Western blot analysis of cell lysates from HMLE/pBP, HMLE/pBP-Twist and HMLE/pBP-Twist/ESRP1 cells probed with antibodies as indicated. α- tubulin was used as a loading control. (B) Still images from a live cell-tracking experiment of cells, as indicated, labeled with a cellular dye CMFDA and plated in the monolayer mixed 1∶20 with unlabelled cells. Cells were tracked for 12 hours. Cell tracks were generated using semi-automated cell tracking and represent single cell tracks over 12 hours with 10 minutes intervals. Centroids of fluorescent cells are indicated by grey circles. Videos are provided as Videos S1, S2, S3. Windrose plots of the range of motion of individual cells of each cell type are shown next to still images from a live-cell imaging experiment, as indicated. Windrose plots were generated by placing starting points of all cell tracks obtained in the cell tracking experiment into the same spot. (C) The box plot depicts speed distribution of individual cells inferred from live-cell imaging of cells in (A) and analyzed by the Imaris software. Edges of the boxes indicate 25^th^ and 75^th^ percentile and the whiskers 5^th^ and 95^th^ percentile. The line in the box indicates the median of the distribution. n = 138 cells for HMLE/pBP; n = 125 cells for HMLE/pBP-Twist; n = 113 cells for HMLE/pBP-Twist/ESRP1-EGFP. *** = p<0.001.

By far the most strongly downregulated RBPs in EMT were the related factors ESRP1 and ESRP2 (Figure 2B; Table S4). These factors have been proposed to promote an epithelial phenotype by facilitating epithelial-specific splicing of a number of genes, some of which have well documented and essential roles in EMT [24], [41]. Silencing of ESRP1/2 in epithelial cells induced N-cadherin expression without affecting E-cadherin levels and led to a slight, but significant, increase in the rate of monolayer wound healing [28]. We hypothesized that expression of ESRP1 in mesenchymal cells would convert a portion of the mesenchymal splicing program to an epithelial state and allow us to examine the role of alternative splicing in the context of Mesenchymal-to-Epithelial Transition (MET). We introduced ESRP1-EGFP into HMLE/pBP-Twist cells, immortalized human mammary epithelial cells that ectopically express Twist [18], and analyzed expression of canonical EMT markers. As expected, control HMLE/pBP epithelial cells expressed high levels of E-cadherin while HMLE/pBP-Twist mesenchymal cells expressed high levels of N-cadherin (Figure 6A; [18]). Expression of ESRP1 in HMLE/pBP-Twist cells was sufficient to switch ENAH splicing to an epithelial pattern, as evident by the inclusion of epithelial-specific 11a exon of ENAH (Figure 6A). However, ESRP1-expressing cells still had high levels of N-cadherin and low levels of E-cadherin. Thus, ESRP1 expression is sufficient to alter splicing of some targets but is not sufficient to alter expression of EMT markers in mesenchymal cells.

One important consequence of EMT is altered cell migration. To assess the effect of ESRP1 expression on cell migration qualitatively, we analyzed cell the movement of cells migrating out of a matrigel drop by time-lapse microscopy. This assay is similar to a standard *ex vivo* EMT assay used in the studies of developmental EMT to assess cell migration of endocardial cushion explants [42]. Cells were reconstituted in a small volume of matrigel and allowed to migrate out of the cell-matrigel drop for 24 hrs (Figure S7). Almost no difference in migration was observed in 8 hrs between control epithelial cells, mesenchymal cells and the same cells expressing ESRP1. However, by 19 hrs the epithelial HMLE/pBP cells continued to migrate as an epithelial sheet, keeping in tight contact with each other, while HMLE/pBP- Twist mesenchymal cells acquired a spindle-shaped morphology, migrated as individual cells and for a longer distance than epithelial cells during the same time period (Figure S7). Interestingly, HMLE/pBP-Twist cells expressing ESRP1 became elongated but continued to move in contact with each other. These differences in migration were further manifested at 24 hrs, suggesting that ESRP1 expression conferred epithelial-like properties to the migration of mesenchymal HMLE/pBP-Twist cells (Figure S7). To analyze the migration characteristics of mesenchymal cells upon ESRP1 expression quantitatively, we utilized an “in monolayer” migration assay [43] that evaluates the movement of individual cells within a monolayer in contrast to a “sheet monolayer” motility assay which assesses collective cell migration towards an open wound [44]. Epithelial HMLE/pBP cells migrate efficiently only when plated in a monolayer in contact with other cells, while mesenchymal HMLE/pBP-Twist cell movement is attenuated by cell-cell contact (H.D. Kim, FBG and D.Lauffenburger, unpublished observations). Control HMLE/pBP epithelial cells, HMLE/pBP-Twist mesenchymal cells and HMLE/pBP-Twist cells expressing ESRP1-EGFP were labeled with whole-cell tracking dye and plated along with the equivalent unlabeled cell types such that the labeled cells represented 5% of cells within a confluent monolayer to assess migration in the presence of cell-cell contact (Figure S8A). As expected, epithelial cells exhibited significant movement in a 17 hr cell tracking experiment [43], [45], while confluent mesenchymal cells moved a little, if at all (Figure 6B; Videos S1, S2). Surprisingly, upon expression of ESRP1, mesenchymal cells demonstrated significant locomotion, bypassing their typical contact inhibition of motility and instead resembling the movement of epithelial cells in a monolayer (Figure 6B; Video S3). Windrose plots of cell movement, where all cell tracks are placed at the same starting point, clearly demonstrated the extent of motion for each cell type (Figure 6B). While many epithelial HMLE/pBP cells traversed paths of up to 300 µm in length, mesenchymal HMLE/pBP-Twist cells moved less than 100 µm. Interestingly, many ESRP1 expressing mesenchymal cells exhibited intermediate range of motion of about 200 µm (Figure 6B). Analysis of the cell movement parameters revealed that the speed of ESRP1- expressing cells was significantly increased compared to the speed demonstrated by mesenchymal cells without ectopic ESRP1 expression (Figure 6C). The total path and overall displacement of HMLE/pBP-Twist/ESRP1 cells were also increased significantly (Figure S8B). The most likely explanation of this data is that splicing changes resulting from ESRP1 expression are sufficient to shift the migration properties of mesenchymal cells towards an epithelial-like phenotype. However, we cannot rule out indirect effects or possible uncharacterized functions of ESRP1.

**Figure 7:**
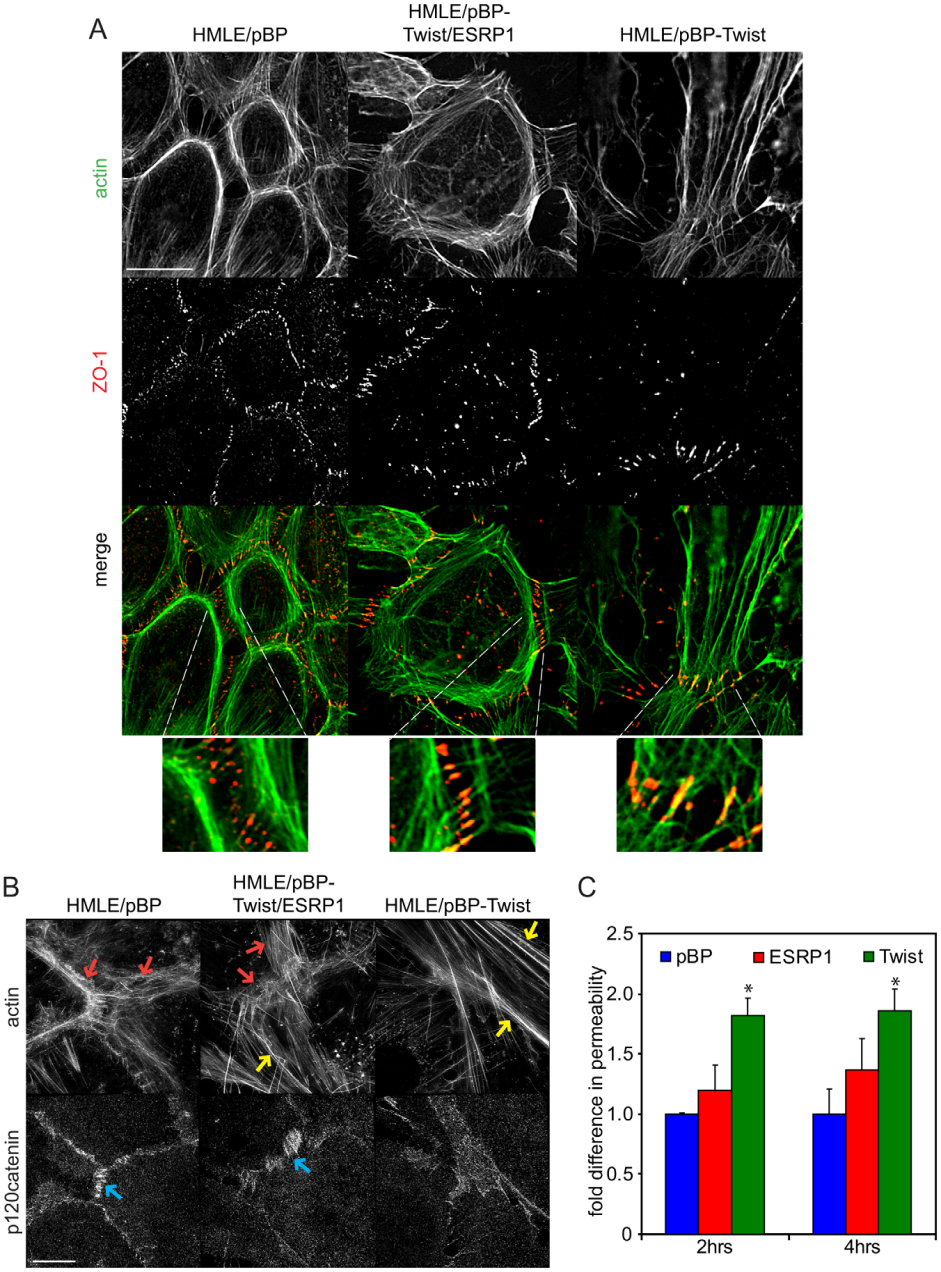
Expression of ESRP1 changes actin organization and localization of junctional markers in mesenchymal cells towards epithelial morphology. (A) Immunofluorescence of cells, as indicated, using anti-ZO-1 antibody and Alexa350-phalloidin. Scale bar, 20 µm. Insets were 5× magnified. (B) Immunofluorescence of cells, as indicated, using anti-p120catenin antibody and Alexa405-phalloidin. Red arrows mark peripheral actin. Yellow arrows mark stress fibers. Blue arrows mark p120catenin at cell junctions. Scale bar, 5 µm. (C) Bar graph depicting movement of Texas Red–dextran across confluent monolayers of HMLE cells, as indicated, at 2 hrs and 4 hrs after addition of dextran compared to control cells expressing pBP (*, P<0.05; *n* = 6). Error bars represent SD.

The actin organization and structure of cell-cell contacts have a substantial effect on the migration of cells within monolayers. To characterize phenotypic changes underlying differences in cell migration behavior of epithelial HMLE/pBP cells, mesenchymal HMLE/pBP-Twist cells, and HMLE/pBP-Twist cells expressing ESRP1, immunofluorescence analysis was used to visualize actin organization and cell-cell junctions (Figure 7A; Figure S9). As expected, three-dimensional structured illumination microscopy revealed the presence of circumferential actin belt in epithelial cells, while actin stress fibers prevailed in mesenchymal cells (Figure 7B). Interestingly, actin organization was altered in mesenchymal cells upon expression of ESRP1. While some stress fibers were present in the central part of the cell, prominent accumulation of peripheral circumferential actin, characteristic of epithelial cell morphology, was also observed. p120catenin, a marker for cell-cell adhesions, decorated areas of cell-cell contact in HMLE/pBP cells, while in HMLE/pBP-Twist cells p120catenin localization was barely visible at cell contact points and could be observed only in areas where adjacent cells overlapped without forming obvious junctions (Figure 7B). Expression of ESRP1 led to increased recruitment of p120catenin to the sites of cell-cell adhesion (Figure 7B). The tight junction marker ZO-1 as well as alpha-catenin localized to actin filaments that perpendicularly terminated at cell-cell borders in immature cell-cell junctions of epithelial cells. In contrast, ZO-1 and alpha-catenin localized to the sites of focal cell-cell contact at the ends of stress fibers in mesenchymal cells (Figure 7A; Figure S9A). Interestingly, expression of ESRP1 in mesenchymal cells led to patterns of ZO-1 and alpha-catenin resembling their localization in epithelial cells. Thus, ESRP1 expression in mesenchymal cells partially reverted actin organization and cell-cell junction morphology towards the epithelial phenotype.

A defining feature of epithelia and endothelia is to separate compositionally distinct fluid phase compartments by providing a barrier to ion and solute passage, a prerequisite for the development of most organ systems in vertebrates [46], [47]. To assess whether the change in actin organization and cell-cell junction morphology in mesenchymal cells upon expression of ESRP1 would have functional consequences, we compared the ability of fluorescently tagged dextran to cross a confluent monolayer of epithelial HMLE/pBP cells, mesenchymal HMLE/pBP-Twist cells and the same cells expressing ESRP1. As expected, permeability of the HMLE/pBP-Twist cell monolayer was almost two-fold higher than permeability of the HMLE/pBP cell monolayer (Figure 7C). Strikingly, expression of ESRP1 in HMLE/pBP-Twist cells increased their barrier function significantly, resulting in permeability that was less then 1.5 fold higher than control epithelial cells (Figure 7C). Thus, expression of ESRP1 lead to a substantial increase in barrier function of mesenchymal cells, caused by the epithelial-specific splicing induced in mesenchymal HMLE/pBP-Twist cells. These results suggest that ESRP1-mediated splicing changes may drive epithelial-like re-organization of peripheral actin and cell-cell junctions that underlie barrier function.

### Depletion of RBFOX2 in mesenchymal cells leads to a partial reversion towards epithelial phenotype

As noted above, our analysis along with published data [26] suggest that the RBFOX2 splicing factor likely controls a substantial subset of EMT-dependent alternative splicing (Figure 2A, 2C). To assess the effect of RBFOX2 depletion on cell phenotype, we treated HMLE/pBP-Twist mesenchymal cells with scrambled shRNA or with shRNA targeting RBFOX2. qRT-PCR analysis demonstrated ∼80% depletion of RBFOX2 mRNA (Figure S10A), while RBFOX2 protein levels became virtually undetectable (Figure S10C). RT-PCR analysis of the known RBFOX2 targets FAT and PLOD2 [26] confirmed changes consistent with depletion of RBFOX2 activity. In mesenchymal cells treated with RBFOX2 shRNA, FAT alternative exon inclusion was reduced from 40% to 5%. A less dramatic but significant effect on exon inclusion was also observed for the PLOD2 alternative exon (Figure S10B).

Expression of many EMT markers was unaffected by RBFOX2 depletion. No difference in expression was observed for N-cadherin and fibronectin compared to scrambled shRNA-treated control cells (Figure S10C). However, vimentin levels were reduced, indicating a partial loss of the mesenchymal expression program in HMLE/pBP-Twist cells upon RBFOX2 knockdown. Immunofluorescence analysis revealed that RBFOX2 depletion in mesenchymal HMLE/pBP-Twist cells shifted their morphology from spindle-shaped to cobblestone-like, resembling epithelial cell morphology (Figure S10D). Stress fibers, prominent in HMLE/pBP-Twist cells, were not readily observed after RBFOX2 depletion. Junctional markers like ZO-1, p120catenin and alpha-catenin brightly decorated cell-cell contacts, suggesting that cell junctions were formed in these cells in contrast to HMLE/pBP-Twist mesenchymal cells, where these markers were barely visible at sites of cell-cell contact (Figure S10D). Qualitative assessment of cell migration properties using a matrigel drop assay described above demonstrated that HMLE/pBP-Twist cells expressing a scrambled shRNA exhibited an individual cell migration pattern and scattered in 24 hrs of plating characteristic of mesenchymal cells. In contrast, cells expressing RBFOX2 shRNA migrated as a sheet, staying in contact with each other (Figure S10E). Together, these data suggests that, similar to ectopic ESRP1 expression, knockdown of RBFOX2 conferred a number of epithelial features to mesenchymal cells, presumably by shifting their splicing program from mesenchymal to partially epithelial.

## Discussion

In the present study, we profiled the transcriptome of human mammary epithelial cells induced to undergo EMT by activation of Twist, a transcription factor important for EMT induction during embryonic development and metastasis. Using this system, we observed an EMT-associated global change in alternative splicing of a number of genes that are involved in functions crucial for EMT progression, such as cell adhesion, cell motility, and cytoskeletal remodeling. Several of the splicing changes discovered *in vitro* were also found to occur in a panel of breast cancer cell lines and *in vivo* in primary human breast cancer samples. We also demonstrated that expression of an epithelial specific splicing factor, ESRP1, was sufficient to cause a substantial shift in the actin organization, migration properties and barrier function of mesenchymal cells towards the epithelial phenotype, while depletion of the splicing factor RBFOX2 also conferred some epithelial properties to mesenchymal cells. Altogether, the present evidence leads us to propose that alternative splicing plays a major role in EMT and tumor progression by changing alternative isoform expression of genes important for epithelial and mesenchymal cell morphology and motility.

### Changes in alternative splicing contribute to pathological EMT

Transcriptional regulation of EMT has been a focus of numerous studies in cancer cell lines and primary tumor samples in the last decade [15]. A number of transcription factors have been identified that repress key regulators of EMT such as E-cadherin, and induce transcription of the drivers of mesenchymal phenotype, including N-cadherin and vimentin [16], [18], [48], [49]. Changes in alternative isoform expression during EMT have been observed previously only for a handful of genes including FGFR2, p120catenin, ENAH and CD44 [22]–[25], [50]. Recently, epithelial-specific splicing factors ESRP1 and ESRP2 have been shown to regulate splicing of a subset of genes that contribute to the epithelial phenotype [28]. However, the extent to which coordinated changes in splicing might contribute to phenotypic and morphological changes during EMT has not been investigated systematically. Our results demonstrate that thousands of genes undergo changes in alternative isoform expression during EMT, establishing the existence of a program of alternative RNA processing accompanying EMT.

Many of the alternative splicing events we observed may have a major effect on protein functions important for EMT, including regulation of cell migration, cell adhesion and actin cytoskeleton remodeling (See Figure 5B; Table 1). For example, inclusion of alternative exon in the C-terminus of ARHGEF11, a Rho guanine nucleotide exchange factor (GEF) 11, also known as PDZ-RhoGEF, is increased in mesenchymal cells. Interestingly, removal of the C-terminus of ARHGEF11 results in a remarkable increase in its ability to induce RhoA activation *in vivo* and promotes neoplastic transformation [51]. Furthermore, components of key pathways that control cell motility, invasion and EMT itself are affected by alternative splicing (Table 1), including components of the Wnt and TGF-β signaling pathways. Some RNA regulatory proteins were also affected. For example, increased inclusion of exon 5 of the splicing factor MBNL1 was detected in epithelial cells, a change that occurs in models of myotonic dystrophy and alters the intracellular localization of the protein from cytoplasmic to nuclear [52]–[54]. Interestingly, several previously uncharacterized mRNA isoforms of genes that control important aspects of EMT have been found in this analysis. For example, a 40% increase in inclusion of a 26 aa region in SCRIB (a homolog of *Drosophila* scribble), involved in regulation of apical-basal polarity and directional migration of epithelial cells [55], [56], was observed in mesenchymal cells that might alter a PKC phosphorylation site. This suggests that a cDNA containing this 26 aa exon may not encode the appropriate isoform to use when studying the function of SCRIB in epithelial cells. Altogether, our analysis demonstrates that alternative splicing in EMT leads to changes in protein functions in ways that contribute to the establishment of mesenchymal phenotype, and identifies many widely studied molecules with the potential for significant isoform-dependent functions during EMT.

**Table 1:** Functional consequences of alternative splicing in EMT.

*Gene*	*EMT-relevant gene functional tendency*	*Change in the reading frame*	*dΨ*	*RNA and/or protein region altered by alternative splicing*	*Inclusion isoform expressed in*
	**Regulation of actin cytoskeleton and cell adhesion**				
WASF1	regulation of actin cytoskeleton	alternative first exon (AFE)	−0.37	5′UTR	epithelial
VCL	stabilization of E-cadherin at adherens junctions^3^	intron retention (RI)	−0.22	5′UTR	epithelial
ABI-2	formation and stability of cell junctions^16^	inframe A3SS	−0.21	homeo-domain homologous region	epithelial
PTPRF	stabilization of adherens junctions^1^	inframe inc/del (SE)	−0.13	FNIII-like domain 5 (LASE-c)^2^	epithelial
ILK	tumor invasion via inhibition of E-cadherin^15^	inframe A3SS	0.34	5′UTR	mesenchymal
ABL2	regulation of actin remodeling^20^	alternative first exon (AFE)	0.39	N-terminal	mesenchymal
SCRIB	tumor suppressor; supports epithelial cell polarity^24^	inframe inc/del (SE)	0.39	partially affects PKC phosphorylation motif	mesenchymal
CTNND1	cell adhesion and signal transduction	inframe inc/del (SE)	0.68	N-terminal RhoA binding stabilization domain	mesenchymal
	**Induction of EMT**				
FGFR2	induction of EMT^7^	MXE	−1	IgIII - like domain	epithelial
FGFR1	induction of EMT^8^	MXE	−0.54	IgIII - like domain	epithelial
STX2	epithelial cell morphogenesis and activation^6^	premature Stop	−0.46	C-terminal	epithelial
VEGFA	induction of EMT^12^	intron retention (RI)	0.4	3′UTR	mesenchymal
TEAD1	transcriptional activation of mesenchymal targets^4^	inframe inc/del (SE)	0.61	internal repeat downstream of TEA domain^5^	mesenchymal
	**Cell motility and invasion**				
FAT1	enhancement of cell migration and invasion^9^	inframe inc/del (SE)	0.38	cytoplasmic domain (FAT1+12^10^)	mesenchymal
PPFIBP1	tumor cell motility and migration^11^	inframe inc/del (SE)	0.42	phosphorylation motif for Akt1	mesenchymal
NF2	tumor suppressor; inhibitor of cell migration^23^	alternative first exon (AFE)	0.37	N-terminal intermolecular association domain	mesenchymal
	**TGF-beta pathway**				
E2F4	mediator of TGF-beta response^14^	alternative last exon (ALE)	0.24	C-terminal	mesenchymal
SMAD2	mediator of EMT induction via TGF-beta pathway^13^	alternative first exon (AFE)	0.4	N-terminal	mesenchymal
BMP1	promotion of tumor cell migration^21,22^	inframe inc/del (SE)	−0.13	PKA C-terminal phosphorylation site	epithelial
	**Wnt signaling pathway**				
DKK3	Wnt signaling antagonist^19^	alternative first exon (AFE)	0.36	5′UTR	mesenchymal
CSNK1A1	promotes epithelial cell-cell adhesion^18^	A5SS	0.43	elongated C-terminus	mesenchymal
CSNK1G3	Wnt pathway regulation	inframe inc/del (SE)	0.55	C-terminal	mesenchymal

Column 1, EMT-relevant genes that are alternatively spliced; Column 2, EMT-related function of the corresponding protein; Column 3, the kind of alternative splicing event; Column 4 indicates the change in the amount of the inclusion isoform (dΨ = Ψ(mes)−Ψ(epi)); Column 5 describes RNA region or a known protein domain affected; Column 6 indicates whether inclusion isoform is expressed in epithelial or mesenchymal cells.

Could key aspects of EMT and/or MET be driven by splicing changes alone, independent of the transcriptional machinery? Recent data suggests that systemic dissemination of tumor cells occurs at early stages of tumor development [57], therefore, targeting MET therapeutically might prove more effective since at the time of diagnosis it may already be too late to successfully target EMT-inducing events. Thus we chose to assess contribution of changes in alternative splicing to MET. Our experiments with ESRP1 and RBFOX2 splicing factors suggest that epithelial splicing induced in mesenchymal cells by expression of ESRP1 or the loss of mesenchymal splicing resulting from depletion of RBFOX2 are not sufficient to convert gene expression into an epithelial pattern. However, mesenchymal cells expressing ectopic ESRP1 or depleted of RBFOX2 exhibited actin organization, barrier function and migration characteristics shifted significantly towards an epithelial phenotype, indicative of a partial MET. Our data along with other reports [58], [59] suggest that although transcriptional control is extremely important to drive EMT, alternative splicing is required to execute the complex changes needed for cells to undergo the dramatic phenotypic change from epithelial to mesenchymal states.

Comparison of EMT-dependent skipped exon events identified in the current study to ESRP-regulated ones [28], [41] revealed that out of ∼1500 EMT-dependent events (FDR<0.05), only 116 seem to be regulated by ESRP1,2 (Figure S11A). Interestingly, ESRP1 expression in clinical samples correlated with the inclusion of an alternative exon of ENAH but no correlation was observed with the presence of lymph node metastasis (Figure S11B). PTB and RBFOX2 may control a number of EMT-driven splicing events, as evident by a significant overlap between exons associated with CLIP-Seq tags and exons that undergo splicing changes during EMT. However, ESRP1, RBFOX2 and PTB together may regulate only a fraction of all EMT-associated alternative splicing (Figure 2A, 2C; Figure S11A), so it is likely that other splicing factors also play important roles in executing the EMT splicing program. Our RBP motif enrichment analysis suggests involvement of the MBNL family of splicing factors and several hnRNP proteins, including hnRNPs F/H, L and PTB. Potentially, alteration of a combination of ESRP1, RBFOX2 and/or other specific splicing factors could be sufficient to drive many phenotypic aspects of EMT. In other words, epithelial cells might potentially bypass the traditional EMT-inducing transcriptional networks to acquire mesenchymal-like phenotypes, when triggered by global changes in splicing programs that enable an EMT-like transformation. This raises the intriguing possibility that instances where invasion and metastasis occurs without changes in canonical EMT expression markers may arise from splicing-driven phenotypic changes.

### EMT in primary breast cancers

Evidence for EMT in clinical carcinomas has been difficult to obtain, leading to a controversy regarding the role of EMT as a prerequisite for metastasis. Although EMT and MET have been observed in the animal model of prostate cancer [60], the presence of regions of well-differentiated epithelial morphology within some invasive primary tumors and metastatic lesions appears to conflict with a role for EMT in metastatic progression [7]. A number of factors that may account for this discrepancy have been suggested, including: 1) incomplete EMT may be sufficient for cells to metastasize; 2) EMT might only occur in a small number of cells within the tumor mass that would quickly disappear by intravasating into blood or lymphatic vessels; and, 3) after colonization, tumor cells revert to an epithelial morphology at metastatic sites through a reciprocal process of mesenchymal to epithelial transformation (MET) [13], [15]. Thus, clinical samples of primary tumor and metastatic nodules often do not show evidence of EMT because the relevant cells display a mesenchymal phenotype only when they are in transit from the primary tumor to the site of mestastasis. Moreover, if indeed only a few cells in the primary tumor undergo EMT prior to migration, RNA from these cells would be diluted by RNA from the luminal parts of the tumor in qRT-PCR analyses. FNA samples seem to be an attractive alternative to assess EMT. FNA of some IDCs, where many cells are loosely attached to the tumor mass, collect motile cells that may already be ‘in transit’ from the primary tumor to secondary sites, some of which might presumably have undergone EMT.

In our analysis of EMT-associated splicing changes in IDCs from breast cancer patients collected by FNA, we identified two groups of IDCs. In one group, inclusion of a set of epithelial splicing events was observed; while in a second group inclusion of mesenchymal splicing events was detected, suggesting a post-EMT phenotype. These data indicate that in some of the IDCs, tumor cells underwent EMT, consistent with the idea that EMT is associated with, and can contribute to cancer progression. We hypothesize that IDCs where mesenchymal splicing events were identified are more likely to metastasize than tumors exhibiting the epithelial splicing pattern, since recent studies suggest that expression of EMT program is associated with poor clinical outcome in some tumor types [61], [62].

### EMT–associated alternative splicing events as potential prognostic and diagnostic markers for breast cancer metastasis

Splicing aberrations have been associated with several diseases, including cancer, where altered splicing can lead to production of protein isoforms with oncogenic properties [63]. A large-scale analysis of alternative splicing in ductal breast tumors of 600 cancer-associated genes identified 41 breast cancer-specific markers that discriminate between normal breast tissue and ductal breast tumors [64]. A number of shared splicing events have been recently demonstrated in a panel of breast and ovarian cancers using a high throughput RT-PCR approach [65]. Exon array analysis was recently used to identify subtype-specific alternative splicing events in a panel of breast cancer cell lines [26]. Therefore, it appears likely that alternative splicing analysis will dramatically increase the pool of potential biomarkers for cancer diagnostics.

Since EMT is considered an early event in the metastatic process, splicing changes associated with EMT in particular have the potential to become useful prognostic and diagnostic markers for breast cancer metastasis. Analysis of the EMT-driven splicing events in the NCI-60 panel of breast cancer cell lines [39] demonstrated that many of the EMT-associated alternative isoforms are expressed. Furthermore, luminal and basal B cell lines could be distinguished based solely on their splicing patterns, suggesting that EMT-associated alternative splicing events may serve as useful markers for classification of breast cancer cell lines and potentially of human cancers. Moreover, we identified splicing events that might be considered novel markers of EMT *in vivo*. Alternative splicing of ENAH, MLPH, ARHGEF11, MBNL1, FLNB and SLC37A2 transcripts have been confirmed in a number of IDC FNAs, suggesting that our EMT-associated splicing signature may have a prognostic or diagnostic potential. However, a mesenchymal splicing pattern did not correlate with the presence of lymph node metastasis. This finding is not surprising since FNA samples were obtained from recently diagnosed cancer patients and no follow up information is available regarding a possible relapse or metastatic status of the tumor. Therefore, we cannot draw a meaningful conclusion about the overall relevance of the splicing pattern in relation to outcome or presence of lymph node metastasis. Although lymph node status is considered an independent prognostic factor for relative survival of breast cancer patients (National Cancer Institute website), the presence of regional lymph node metastases does not always correlate with subsequent distant spread possibly because the mechanisms of hematogenous spread are different from those for lymphatic spread [66]. Within the clinical groups created using tumor size and lymph node involvement, there is a spectrum of disease behavior. Even patients with stage I lymph node negative breast cancer have 15–25% chance of developing distant metastasis, so breast cancers of early stage must be composed of mixed phenotypes that cannot be stratified using standard approaches such as lymph node status, or tumor size [67]. Thus, an EMT splicing signature may help to stratify early stage breast cancers, however additional studies will be required to determine the prognostic potential of EMT-associated splicing events that we have validated in FNA samples.

A growing body of evidence suggests that EMT is responsible for acquisition of therapeutic resistance by cancer cells [13]. EMT has been implicated in the generation of cancer cells with stem-like characteristics that have a high tumor-initiating potential [68]. Cancer stem cells have been found enriched in residual breast tumors after chemo- or endocrine therapy [68], in colorectal cancer cells after oxaliplatin treatment [69], or in ovarian carcinoma cells after exposure to paclitaxel [70]. Clinical evidence suggests that expression of mesenchymal markers is increased in breast tumors after letrozole or docetaxcel treatment [68]. This indicates that EMT-associated alternative splicing events that we confirmed in FNA samples from patients with IDCs may potentially become predictive biomarkers that can be used for patient selection and/or provide information early during therapy. Further studies specifically designed to identify alternative splicing markers that reflect distinct breast cancer biology in relation to clinical outcomes and prognoses show promise to improving our understanding of EMT and breast cancer at the molecular level.

## Materials and Methods

### Ethics statement

MIT Committee on the Use of Humans as Experimental Subjects

To: Frank Gertler

From: Leigh Firn, Chair

COUHES

date: 04/16/2009

Committee action: Exemption granted

COUHES protocol#: 0904003185

Study title: Gene Expression and splicing analysis during cancer progression.

The above referenced protocol is considered exempt after review by MIT Committee on the use of Humans as experimental subjects pursuant to Federal Regulations 45 CFR Part 46, 101(b)(4).

### Cell culture

Immortalized human mammary epithelial cells (HMLEs) expressing either the empty pBabe puro vector (pBP), pBP-Twist or pWZL-Twist-ER were obtained from Robert Weinberg's laboratory at the Whitehead Institute for Biomedical Research (Cambridge, MA) and cultured as described previously [71]. 4-hydroxy tamoxifen (4-OHT) treatment was performed as described previously [29]. Other plasmids used in this study, procedures used to produce virus, the procedure for the infection of target cells, and the derivation of different cell lines are provided as Text S1.

### Antibodies, Western blotting, and immunofluorescence

Cells were lysed in the presence of 50 mM Tris, pH 8.0, 150 mM NaCl, 0.1% SDS, 0.5% Na-Deoxycholate and 1.0% NP-40 on ice. Twenty micrograms of total protein from each sample were resolved on an 8%–10% SDS-PAGE Gel with Laemmli Running Buffer and transferred to PVDF membranes. The blots were then probed with various antibodies, such as anti-Mena, and anti-Mena-11a, anti-E-cadherin (BD Transduction), anti-Fibronectin (BD Transduction), anti-vimentin V9 (NeoMarkers), or anti-N-cadherin (BD Transduction). Detailed immunofluorescence methods are provided in the Text S1.

### cDNA library preparation for Illumina sequencing

Total RNA was extracted from untreated HMLE/Twist-ER cells (epithelial sample) and after prolonged 4-OHT treatment (mesenchymal sample) using RNeasy Plus Mini kit (Qiagen). Poly-T capture beads were used to isolate mRNA from 10 mg of total RNA. mRNA was fragmented and used for a first-strand cDNA synthesis by random hexamer-primed reverse transcription and subsequent second-strand cDNA synthesis. Sequencing adaptors were ligated using the Illumina Genomic DNA sample prep kit. Fragments 200 bp long were isolated by gel electrophoresis, amplified by 16 cycles of PCR, and sequenced on the Illumina Genome Analyser, as described previously [20].

### Computational analyses of RNA–Seq, exon array data, motif analysis, and clustering

Computational and statistical methods are described in the Text S1. Briefly, for analysis of RNA-seq data, reads were mapped to the union of the genome and a database of junctional sequences derived from AceView/Acembly annotation. Expression analysis was based on reads that were mapped to constitutive exons among annotated RefGene transcripts of each gene. Splicing analysis was based on read density supporting either isoforms of an alternative splicing event from a database of alternative isoform events. For more details see the Text S1. Alignment and raw sequencing reads were deposited in Gene Expression Omnibus with accession number GSE30290.

### Reverse transcriptase PCR analysis

Total RNA for validation of splicing events in HMLE/Twist-ER cells was extracted using RNeasy Plus Mini kit (Qiagen) and reverse transcribed with Superscript II (Invitrogen). The resulting cDNA was used for 25 cycles of PCR with primers listed in the Text S1. Then samples were subjected to 10%TBE gel electrophoresis (Bio-Rad), stained with SYBR Safe DNA Gel Stain (Invitrogen), scanned (Typhoon, GE Healthcare) and quantified (ImageQuant 5.2). Total RNA from FNA samples was extracted using RNeasy Plus Micro kit (Qiagen). The resulting cDNAs were used for qPCR analysis using iQ Syber-Green Supermix (BioRad) in triplicates. qPCR and data collection were performed on iCycler (BioRad). Primer sequences used to amplify cDNAs and the detailed description of quantification analysis are listed in the Text S1.

### Human tissue selection and FNA biopsy procedure

Lumpectomy and mastectomy specimens that arrive to grossing rooms at AECOM hospitals Montefiore and Weiler for pathological examination were used for tissue collection. The specimens were sectioned as usual at 0.5 or 1.0 cm intervals to locate and visualize the lesion of interest. Four to 5 FNA aspiration biopsies (passes) were performed on grossly visible lesions using 25 gauge needles. When an FNA needle is inserted into a malignant tumor it preferentially collects loose tumor cells, as can be noted on FNA obtained smears in Figure 5 and Figure S5. A small number of other cell types may also be present, most commonly inflammatory cells and macrophages. The aspirated material was collected in the cryo-vials, and to assess the adequacy of the sample, a small portion of the aspirated material was taken out of the vial, smeared on a glass slide, air-dried and stained by standard Diff-Quick protocol. The adequacy of the sample was determined by cytopathologic microscopic examination of the smears. Only samples composed of 95% of either benign or malignant epithelial cells were used in the study. Standard cytopathologic criteria such as cell size, nuclear/cytoplasmic ratio, nuclear contours, cell crowding and cohesiveness of the cells were the major criteria for classification into benign or malignant category. Samples containing a mixture of malignant and benign cells, necrotic cell debris, or more than 5% of inflammatory or stromal cells as determined by cytopathologic microscopic examination were discarded. FNA biopsy samples were immediately snap frozen in liquid nitrogen and stored frozen for RNA isolation followed by a qPCR analysis. Specimens were collected without patient identifiers following protocols approved by the Montefiore Medical Center Institutional Review Board.

### Cell migration assays

Matrigel overlay assay was performed as previously described [42]. 10^5^ cells were mixed with 3.5 mg/ml matrigel and polymerized in a drop on top of the matrigel-covered coverslip. Images of migrating cells at 0, 8 hr, 19 hrs, 24 hrs time points were obtained on a Nikon Eclipse TE200 using a 10× DIC objective. Cell migration assay was performed as previously described [43], [72]. Cells were incubated with CMFDA (Invitrogen) for 10 minutes and seeded overnight. Labeled and unlabeled cells were seeded at a 1∶20 ratio. In 24 hrs, cells were placed on an environment-controlled Nikon TE2000 microscope (Nikon Instruments; Melville, NY) and were imaged every 10-minutes for 12 hrs. Image sequences were analyzed with Bitplane Imaris software (Zurich, Switzerland) using the built-in ‘Spots’ function. 12-hour tracks were generated using the ‘Brownian Motion’ algorithm.

### Permeability assay

HMLE/pBP-EGFP, HMLE/pBP-Twist-EGFP and HMLE/pBP-Twist/ESRP1-EGFP cells were seeded at confluence on polycarbonate transwell membrane inserts (3.0 µm pore size; Falcon 353492) and cultured for 3 d. 70 kD of Texas red–dextran (Invitrogen) was added to the top chamber at 2 mg/ml, and its movement into the bottom chamber was monitored over 4 hrs by spectrophotometer.

## Supporting Information

Figure S1 RNA–Seq analysis and validation. (A) The table and pie charts indicate total read numbers obtained for each sample and efficiency of their mapping to the human genome using AceView annotation. (B) Western blot analysis of cell lysates from HMLE/Twist-ER, MCF7 and T47D probed with antibodies as indicated. (C) Read density in exonic and intronic regions is plotted as a function of RPKM in epithelial and mesenchymal samples. (D) Comparison of DY = Y(Mesenchymal)−Y(Epithelial) value defined by RNA-seq analysis to the DY determined experimentaly by semi-quantitative RT-PCR.
(TIF)

Figure S2 EMT is accompanied by a massive change in gene expression. (A) Scatter plot of gene expression during EMT. RPKM values were plotted for epithelial (x axis) and mesenchymal (y axis) samples. Genes upregulated in the mesenchymal sample are marked by red and purple dots, as indicated. Genes upregulated in the epithelial sample are marked by light and dark green dots, as indicated. Genes whose expression did not change are marked in blue. (B) and (C) Gene ontology enrichment analysis of genes downregulated (B) and upregulated in EMT. Gene ontology ‘biological process’, GO_BP_FAT, annotation is depicted in red on the y axis. KEGG Pathway analysis (http://www.genome.jp/kegg/) annotation is depicted in blue on y axis. Benjamini FDR (−log10) is indicated on the x axis. Vertical dotted line marks Benjamini FDR = 0.05.
(TIF)

Figure S3 Regulation of gene expression is independent from regulation of alternative splicing during Twist-induced EMT. Cumulative Density Function (CDF) plot of the distribution of gene expression changes among genes that are alternative spliced (fg (genes with SE events FDR<0.05, |dPsi|>0.1), red line), and not alternatively spliced during EMT (bg (genes in powerset but not in fg), blue dotted line). Kolmogorov-Smirnov (KS) test p-value = 0.69.
(TIF)

Figure S4 Coherence between NCI-60 array data and EMT RNA-Seq dataset increases for highly changed EMT-associated SE events. A bar graph demonstrating the fraction of coherent events between EMT RNA-seq and a panel of NCI-60 breast cancer cell lines [41] as a function of RNA-seq |ΔΨ| cut-offs. The number of events called significant at the corresponding RNA-seq |ΔΨ| cut-offs and exon array FDR<0.25 [41] is depicted above each column.
(TIF)

Figure S5 FNA samples contain negligible amounts of stromal or inflammatory cells. (A) Cellular composition of 15 IDC FNA samples randomly chosen from the 40 FNA samples analyzed in this study. Relative amounts of ductal carcinoma cells (tumor cells), inflammatory cells, and adipocytes and macrophages (stromal cells) are depicted for each sample. (B) Average cellular composition of 15 IDC FNA samples randomly chosen from the 40 FNA samples analyzed in this study. Average relative amounts of ductal carcinoma cells (Tumor cells), inflammatory cells and adipocytes and macrophages (stromal cells) are depicted. Error bars represent SEM. (C) Two representative images of IDC FNA spread. Red error marks fatty droplet. Black error marks inflammatory cell.
(TIF)

Figure S6 Epithelial and mesenchymal inclusion patterns in IDC FNA samples are negatively correlated. (A) Heatmap of six exon inclusion events in FNA samples. The exon inclusion levels were rescaled into [−1,1] and are depicted as shades of red and green. Gray boxes represent data not available (NA). Sample ID is shown to the right. (B) Pvclust clustering tree of six exon inclusion events in (A). AU p-value (confidence) of each subtree is indicated at each branchpoint. The Pearson distance is shown in a ruler on the left. All AU p-values>0.9, and the main epithelial subtree and mesenchymal subtree achieve AU p-value>0.99 indicating the reliability of the clustering tree.
(TIF)

Figure S7 Comparison of the migration behavior of HMLE/pBP, HMLE/pBP-Twist and HMLE/pBP-Twist/ESRP1 cells. Cells were plated in a matrigel drop on top of a thin matrigel layer and allowed to migrate out of the drop for 24 hrs. Migration was followed using 10× DIC imaging at time intervals after the start of the experiment, as indicated. Red line marks the boundary of the initial matrigel drop. Scale bar, 100 µm.
(TIF)

Figure S8 Monolayer migration assay analysis. (A) Phase contrast images of cells, as indicated, plated in a monolayer for the cell tracking experiment in Figure 6. (B) Box plots depict migration parameters inferred from live-cell imaging experiment of cells in Figure 6 and analyzed by the Imaris software. Edges of the boxes indicate 25^th^ and 75^th^ percentile and the whiskers 5^th^ and 95^th^ percentile. The line in the box indicates the median of the distribution. n = 138 cells for HMLE/pBP; n = 125 cells for HMLE/pBP-Twist; n = 113 cells for HMLE/pBP-Twist/ESRP1-EGFP. *** = p<0.001.
(TIF)

Figure S9 Immunofluorescence analysis of cell-cell junctions in HMLE cells. (A) and (B) Immunofluorescence of cells, as indicated, using anti-alpha-catenin (A) and p120 catenin (B) antibodies and Alexa350-phalloidin. Scale bar, 20 µm. Insets were 5× magnified.
(TIF)

Figure S10 Depletion of RBFOX2 confers epithelial-like properties to mesenchymal cells. (A) qPCR analysis of RBFOX2 levels in HMLE/pBP-Twist cells expressing scrambled shRNA or RBFOX2 shRNA using two different primer pairs. (B) RT-PCR analysis of alternative exon inclusion in FAT and PLOD2 in HMLE/pBP-Twist cells expressing scrambled shRNA or RBFOX2 shRNA, as indicated. E marks excluded isoform, I marks included isoform. (C) Western blot analysis of EMT markers and RBFOX2 expression in scrambled or RBFOX2 shRNA treated cells, as indicated. (D) Immunofluorescence analysis of cell junctions using anti-ZO-1, anti-p120catenin, anti-alpha-catenin antibodies and Alexa-350 phalloidin, as indicated. Scale bar 15 µm. Insets were 5× magnified. (E) Comparison of the migration behavior of HMLE/pBP-Twist cells expressing scrambled or RBFOX2 shRNA. For a detailed description see the legend to Figure S4.
(TIF)

Figure S11 ESRP1,2 regulate a subset of EMT-dependent skipped exon events. (A) Venn diagram showing the overlap of skipped exon events reported in Warzecha et al 2009, 2010 [31], [43], and identified from our EMT RNA-seq dataset (FDR<0.05). 1391 events are unique to EMT RNA-seq dataset, 780 events are unique to the union of Warzecha et al 2009, 2010 [31], [43] datasets, 116 are common to both datasets. The numbers beneath the circles denote the number of events reported in the current study and in Warzecha et al 2009, 2010. (B) Heatmap of the ESRP expression levels and exon inclusion level of ENAH alternative exon. The expression values and exon inclusion levels are rescaled into [−1,1] and depicted as shades of red and green. Sample rows were sorted by ESRP1 expression. Sample ID is shown to the right of the heatplot. Lymphnode metastasis for corresponding samples is shown as red (LN positive) and black (LN negative) circles.
(TIF)

Table S1 Skipped and Mutually Exclusive alternative splicing events with FDR<0.05 and |ΔΨ|≥0.03. Column 1 marks the type of event: SE-skipped exon, MXE- mutually exclusive exon. Column 2 - Gene symbol. Column 3 – Ensembl Gene ID. Column 4 - the chromosome number where the gene is located. Column 5 - DNA strand on which the gene is encoded. Column 6 - exon coordinates of the flanking and alternative exons: for SE events - <upstream flanking exon>, <alternative exon>, <downstream flanking exon>/<upstream flanking exon>, <downstream flanking exon>; for MXE events - <upstream flanking exon>, <alternative exon 1>, <downstream flanking exon>/<upstream flanking exon>,<alternative exon 2>, <downstream flanking exon>. Column 7 - the Ψ of the alternative event in the epithelial (pre-EMT) sample. Column 8 - the Ψ of the alternative event in the mesenchymal (post-EMT) sample. Column 9 - ΔΨ = Ψ(mes)−Ψ(epi). Column 10 – FDR.
(XLS)

Table S2 The 5-mers enriched in foreground set over background set of unchanged exons in 250 nt flanking intronic sequences of skipped exons and upstream and downstream exons (FDR<0.05, ΔΨ>0.1). Annotation details: [] are the two flanking exons, <> is the skipped exon: [inUFSeq] I5 ----- I3 < inMFSeq > I5 ------ I3 [inDFSeq]. Column 1 (Exon) indicates a reference exon of the intronic element analyzed. Column 2 (Element) indicates intronic element analyzed: I5- 5′ sequence of the intron, I3- 3′ sequence of the intron. Column 3 (p-value) - the hypergeometric p-value of the 5mer frequency in foreground over that of the background. Column 4 (FDR) - B-H multiple comparison FDR of the p-value. Column 5 (background rate) - the density of the 5mer in the background (set of unchanged SE events). Column 6 (expected frequency) - the expected count of the 5mer in the foreground given the background rate. Column 7 (foreground rate) - the density of the 5mer in the foreground (set of changed SE events). Column 8 (foreground frequency) - the count of the 5mer in the foreground. Column 8 (word) demonstrates the sequence of the 5mer.
(XLS)

Table S3 The 5-mers enriched in foreground set over background set of unchanged exons in 250 nt flanking intronic sequences of skipped exons and upstream and downstream exons (FDR<0.05, ΔΨ<−0.1). Annotation details: [] are the two flanking exons, <> is the skipped exon: [inUFSeq] I5 ----- I3 < inMFSeq > I5 ------ I3 [inDFSeq]. Column 1 (Exon) indicates a reference exon of the intronic element analyzed. Column 2 (Element) indicates intronic element analyzed: I5- 5′ sequence of the intron, I3- 3′ sequence of the intron. Column 3 (p-value) - the hypergeometric p-value of the 5mer frequency in foreground over that of the background. Column 4 (FDR) - B-H multiple comparison FDR of the p-value. Column 5 (background rate) - the density of the 5mer in the background (set of unchanged SE events). Column 6 (expected frequency) - the expected count of the 5mer in the foreground given the background rate. Column 7 (foreground rate) - the density of the 5mer in the foreground (set of changed SE events). Column 8 (foreground frequency) - the count of the 5mer in the foreground. Column 8 (word) demonstrates the sequence of the 5mer.
(XLS)

Table S4 Expression levels of RNA-binding proteins. Column1 - The Gene symbol. Column 2 - the RPKM value of the gene in the epithelial (pre-EMT) sample. Column 3 - the RPKM value of the gene in mesenchymal sample. Column 4 (Ratio) - the ratio of RPKM values (Mes/Epi).
(XLS)

Table S5 Characteristics of the invasive ductal carcinoma (IDC) samples used for the FNA qPCR analysis. Column 1 – samples number; Column 2- tumor size (cm); Column 3 – greatest diameter of the tumor (cm); Column 4 – tumor grade according to the modified Bloom-Richardson scale (1–9). Differentiation status: M- moderate, P – poor, W-well; Columns 5–8 – growth hormone receptor and lymph node status: ER-estrogen receptor, PR- progesterone receptor, Her2 – EGF receptor, LN – lymph node.
(XLS)

Text S1 Supplementary Experimental Procedures.
(DOC)

Video S1 HMLE/pBP cells migrate efficiently in a monolayer. HMLE/pBP cells were labeled with a cellular dye CMFDA and seeded in a confluent monolayer mixed 1∶20 with unlabelled cells. Cells were imaged for 12 hours with 10 min intervals. Cell tracks were generated using semi-automated cell tracking and represent single cell tracks over 12 hours. Centroids of fluorescent cells are marked by grey circles.
(MOV)

Video S2 Monolayer migration of HMLE/pBP- Twist cells is cell-contact inhibited. HMLE/pBP-Twist cells were labeled with a cellular dye CMFDA and seeded in a confluent monolayer mixed 1∶20 with unlabelled cells. Cells were imaged for 12 hours with 10 min intervals. Cell tracks were generated using semi-automated cell tracking and represent single cell tracks over 12 hours. Centroids of fluorescent cells are marked by grey circles.
(MOV)

Video S3 HMLE/pBP- Twist/ESRP1-EGFP cells demonstrate significant locomotion. HMLE/pBP-Twist/ESRP1-EGFP cells were labeled with a cellular dye CMFDA and seeded in a confluent monolayer mixed 1∶20 with unlabelled cells. Cells were imaged for 12 hours with 10 min intervals. Cell tracks were generated using semi-automated cell tracking and represent single cell tracks over 12 hours. Centroids of fluorescent cells are marked by grey circles.
(MOV)
