# Supplementary figures and images for: An EMT–Driven Alternative Splicing Program Occurs in Human Breast Cancer and Modulates Cellular Phenotype
Source: PLoS Genet. 2011 Aug 18;7(8):e1002218. doi: 10.1371/journal.pgen.1002218 (PMC3158048; doi:10.1371/journal.pgen.1002218)

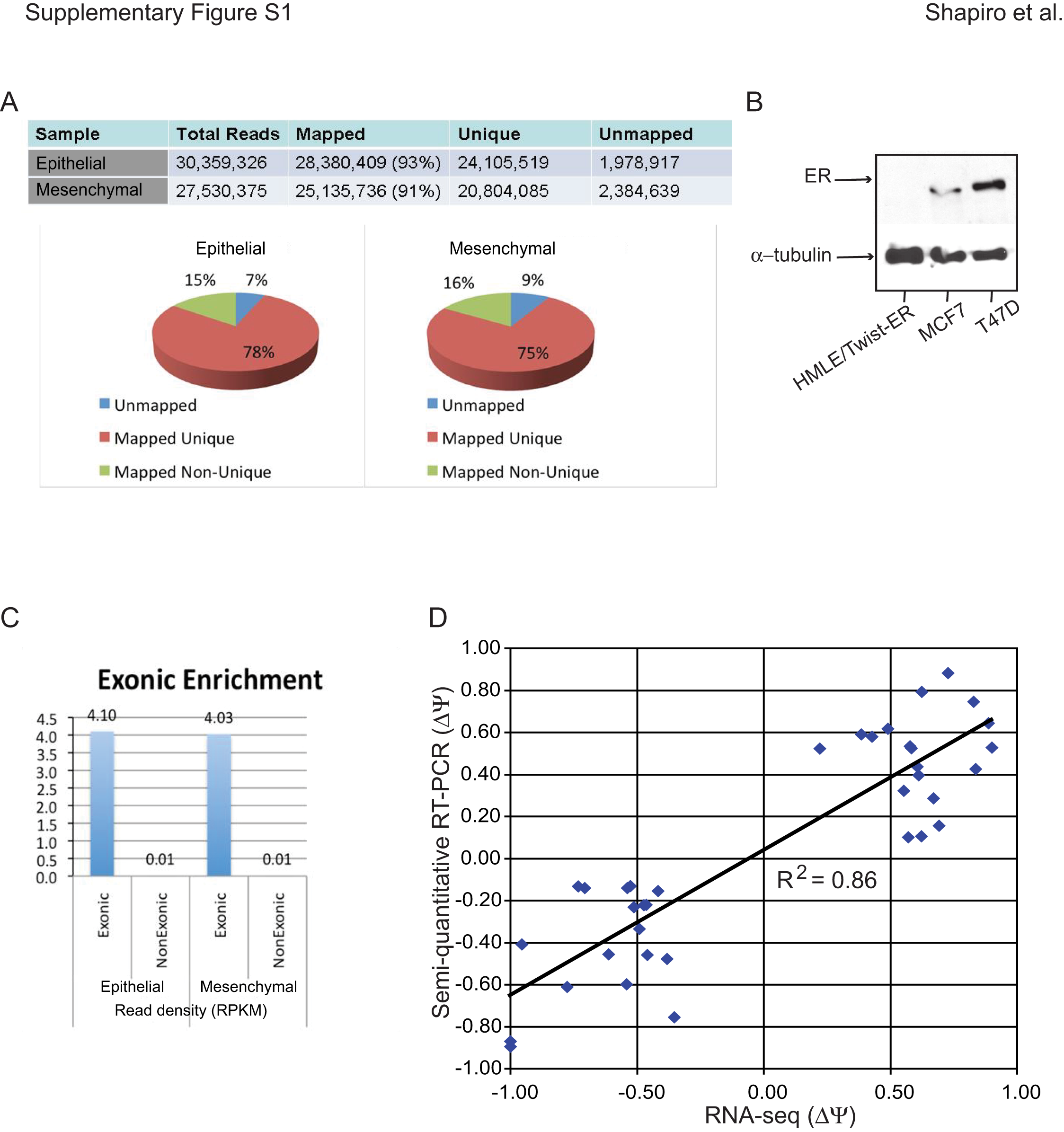

Supplement: Figure S1 — RNA–Seq analysis and validation. (A) The table and pie charts indicate total read numbers obtained for each sample and efficiency of their mapping to the human genome using AceView annotation. (B) Western blot analysis of cell lysates from HMLE/Twist-ER, MCF7 and T47D probed with antibodies as indicated. (C) Read density in exonic and intronic regions is plotted as a function of RPKM in epithelial and mesenchymal samples. (D) Comparison of DY = Y(Mesenchymal)−Y(Epithelial) value defined by RNA-seq analysis to the DY determined experimentaly by semi-quantitative RT-PCR. (TIF) [file pgen.1002218.s001.tif]

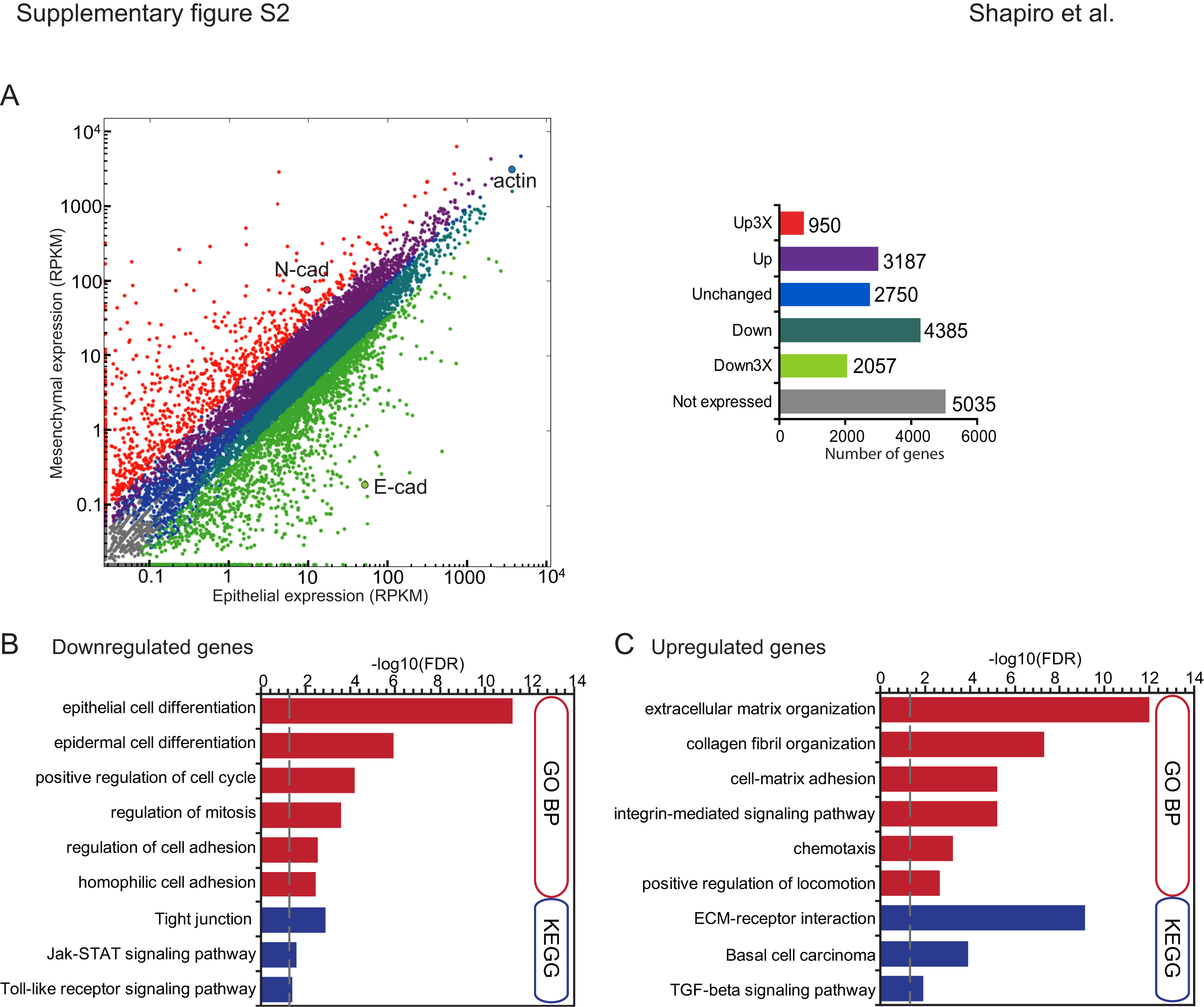

Supplement: Figure S2 — EMT is accompanied by a massive change in gene expression. (A) Scatter plot of gene expression during EMT. RPKM values were plotted for epithelial (x axis) and mesenchymal (y axis) samples. Genes upregulated in the mesenchymal sample are marked by red and purple dots, as indicated. Genes upregulated in the epithelial sample are marked by light and dark green dots, as indicated. Genes whose expression did not change are marked in blue. (B) and (C) Gene ontology enrichment analysis of genes downregulated (B) and upregulated in EMT. Gene ontology ‘biological process’, GO_BP_FAT, annotation is depicted in red on the y axis. KEGG Pathway analysis (http://www.genome.jp/kegg/) annotation is depicted in blue on y axis. Benjamini FDR (−log10) is indicated on the x axis. Vertical dotted line marks Benjamini FDR = 0.05. (TIF) [file pgen.1002218.s002.tif]

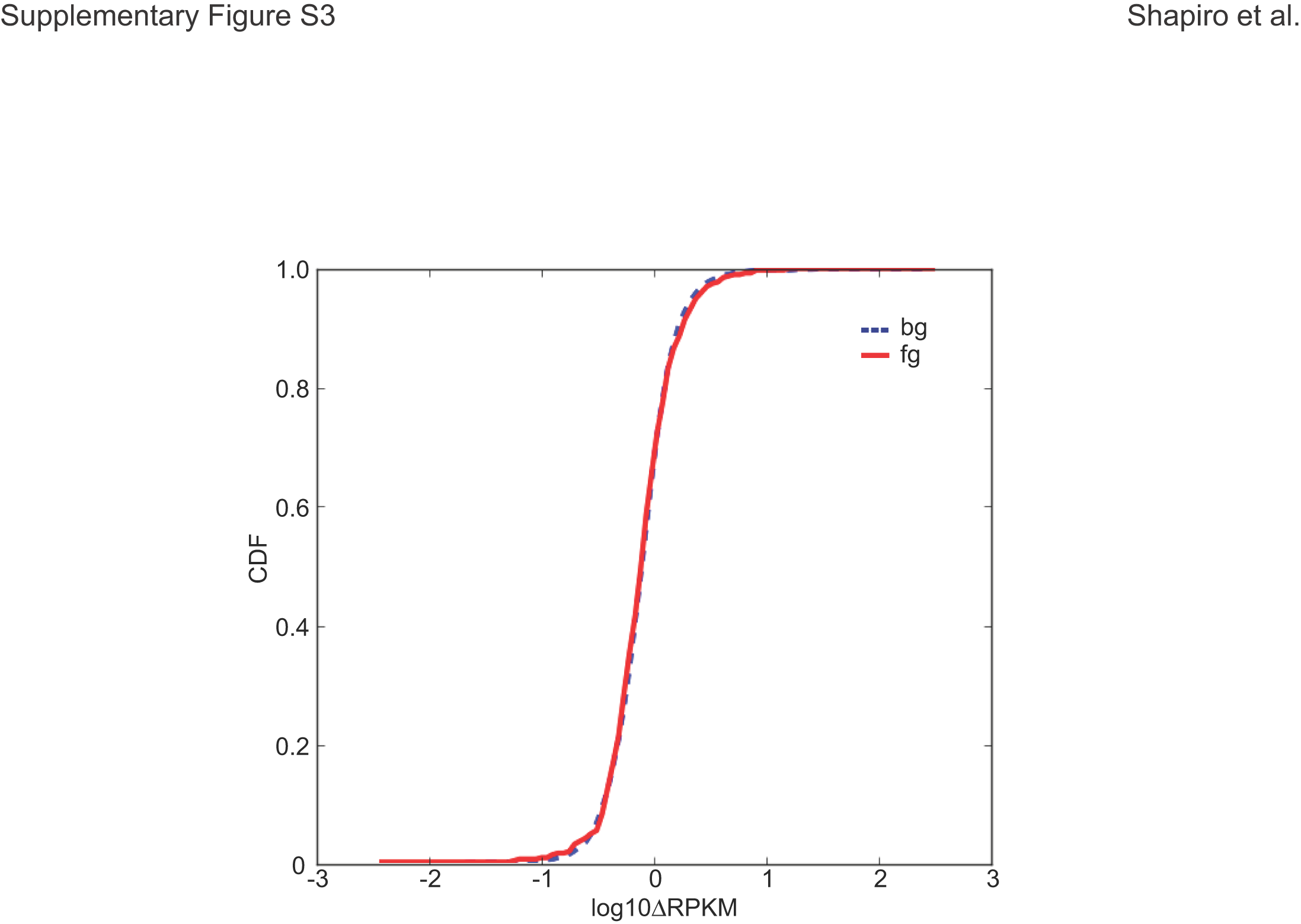

Supplement: Figure S3 — Regulation of gene expression is independent from regulation of alternative splicing during Twist-induced EMT. Cumulative Density Function (CDF) plot of the distribution of gene expression changes among genes that are alternative spliced (fg (genes with SE events FDR<0.05, |dPsi|>0.1), red line), and not alternatively spliced during EMT (bg (genes in powerset but not in fg), blue dotted line). Kolmogorov-Smirnov (KS) test p-value = 0.69. (TIF) [file pgen.1002218.s003.tif]

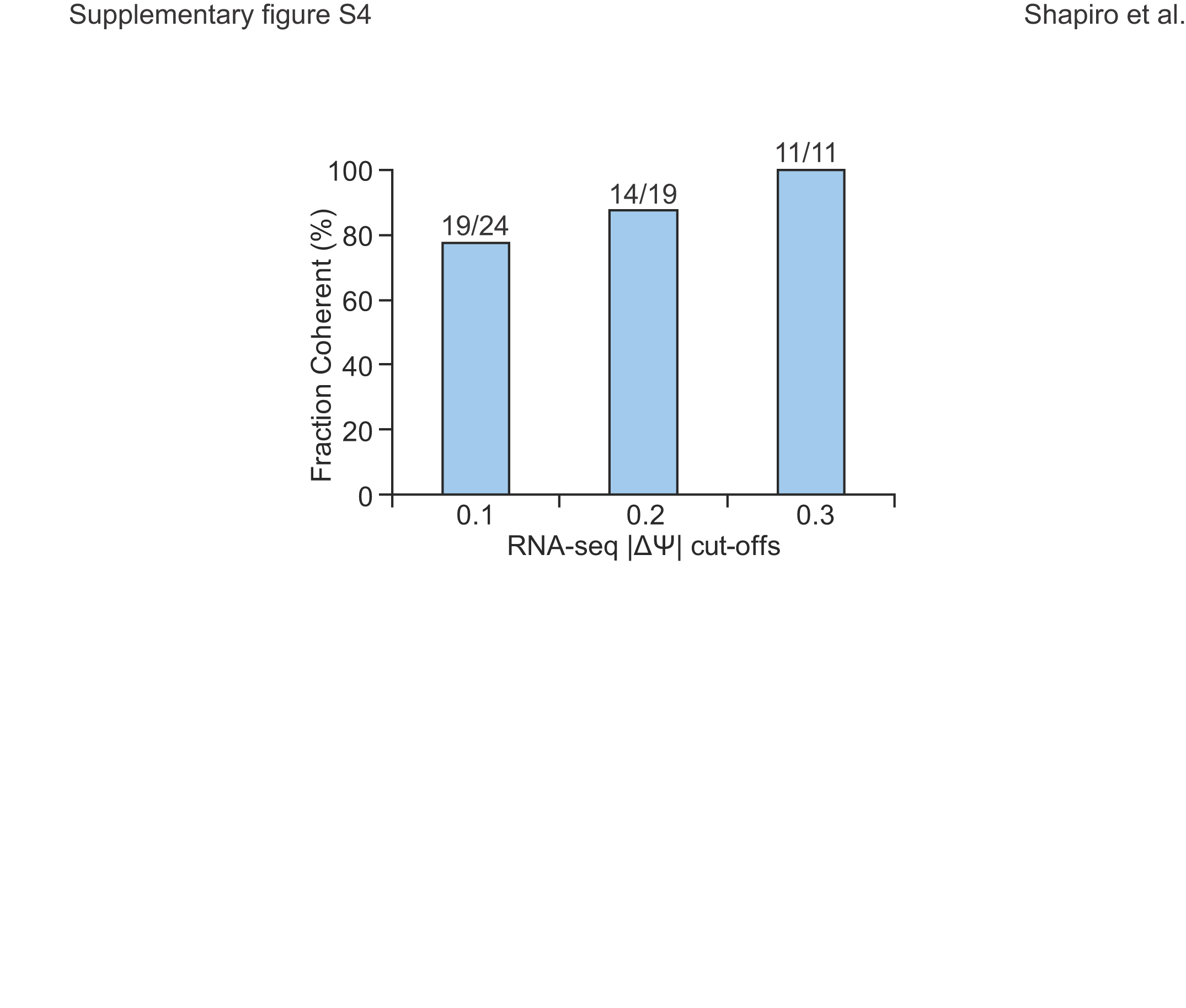

Supplement: Figure S4 — Coherence between NCI-60 array data and EMT RNA-Seq dataset increases for highly changed EMT-associated SE events. A bar graph demonstrating the fraction of coherent events between EMT RNA-seq and a panel of NCI-60 breast cancer cell lines [41] as a function of RNA-seq |ΔΨ| cut-offs. The number of events called significant at the corresponding RNA-seq |ΔΨ| cut-offs and exon array FDR<0.25 [41] is depicted above each column. (TIF) [file pgen.1002218.s004.tif]

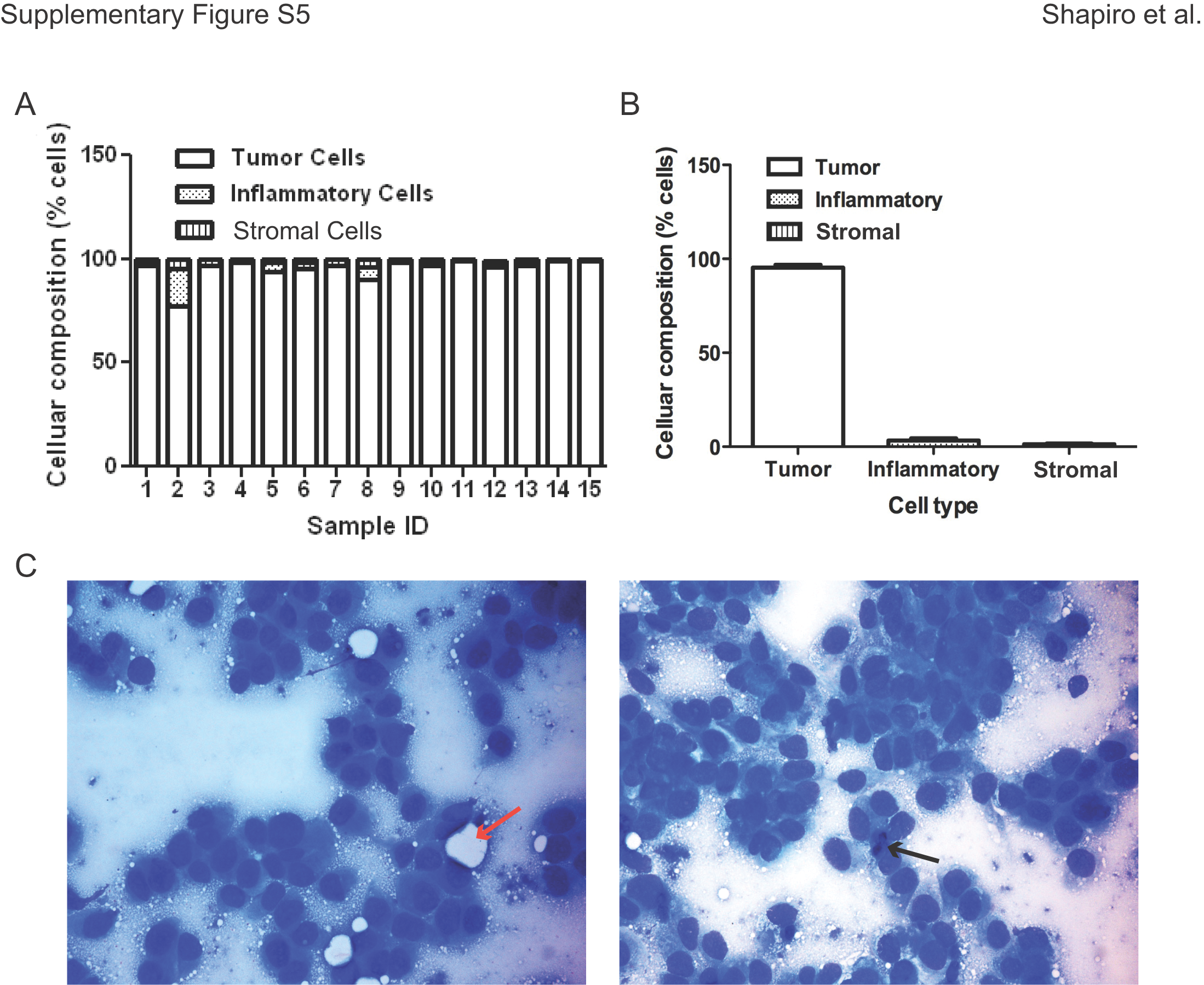

Supplement: Figure S5 — FNA samples contain negligible amounts of stromal or inflammatory cells. (A) Cellular composition of 15 IDC FNA samples randomly chosen from the 40 FNA samples analyzed in this study. Relative amounts of ductal carcinoma cells (tumor cells), inflammatory cells, and adipocytes and macrophages (stromal cells) are depicted for each sample. (B) Average cellular composition of 15 IDC FNA samples randomly chosen from the 40 FNA samples analyzed in this study. Average relative amounts of ductal carcinoma cells (Tumor cells), inflammatory cells and adipocytes and macrophages (stromal cells) are depicted. Error bars represent SEM. (C) Two representative images of IDC FNA spread. Red error marks fatty droplet. Black error marks inflammatory cell. (TIF) [file pgen.1002218.s005.tif]

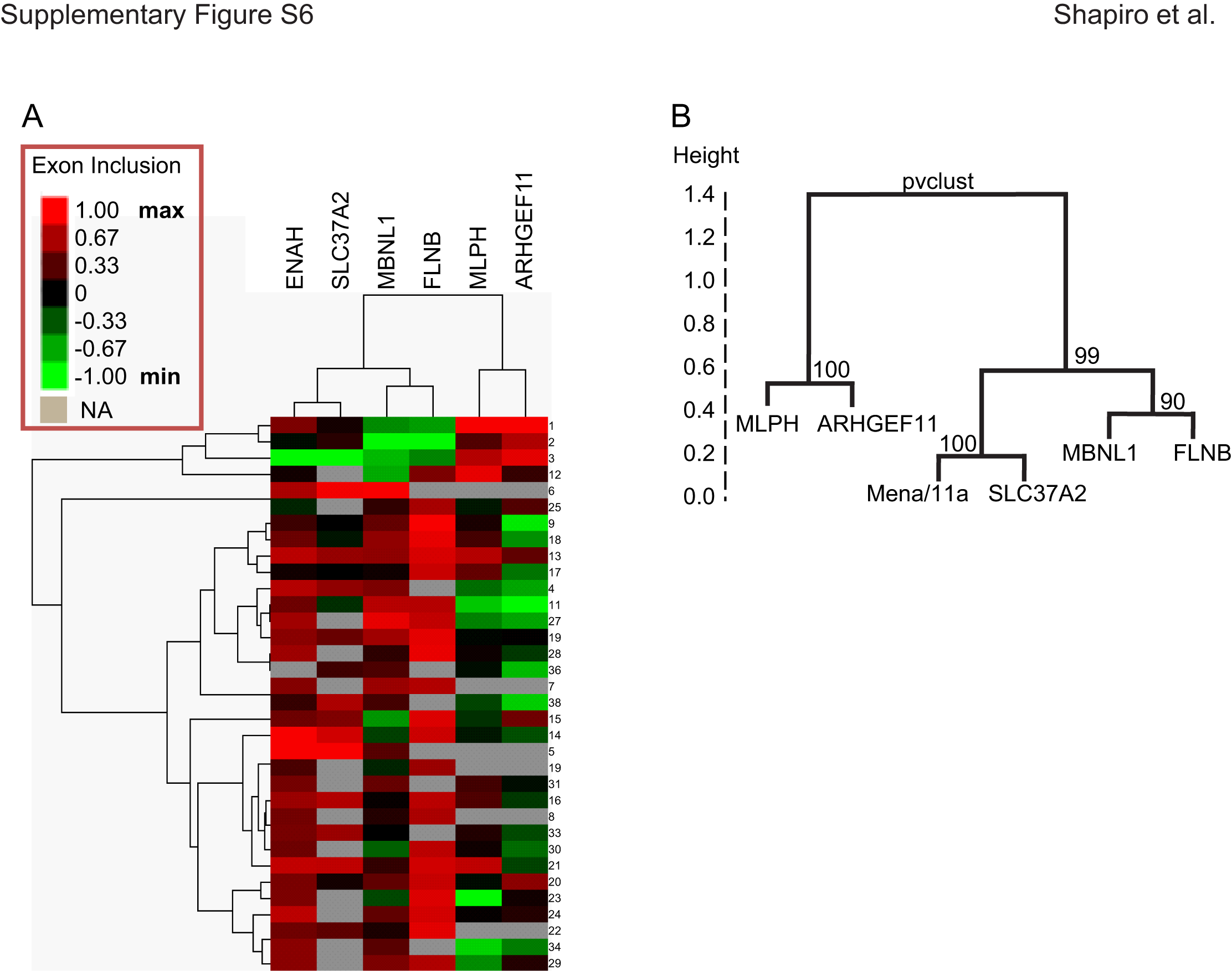

Supplement: Figure S6 — Epithelial and mesenchymal inclusion patterns in IDC FNA samples are negatively correlated. (A) Heatmap of six exon inclusion events in FNA samples. The exon inclusion levels were rescaled into [−1,1] and are depicted as shades of red and green. Gray boxes represent data not available (NA). Sample ID is shown to the right. (B) Pvclust clustering tree of six exon inclusion events in (A). AU p-value (confidence) of each subtree is indicated at each branchpoint. The Pearson distance is shown in a ruler on the left. All AU p-values>0.9, and the main epithelial subtree and mesenchymal subtree achieve AU p-value>0.99 indicating the reliability of the clustering tree. (TIF) [file pgen.1002218.s006.tif]

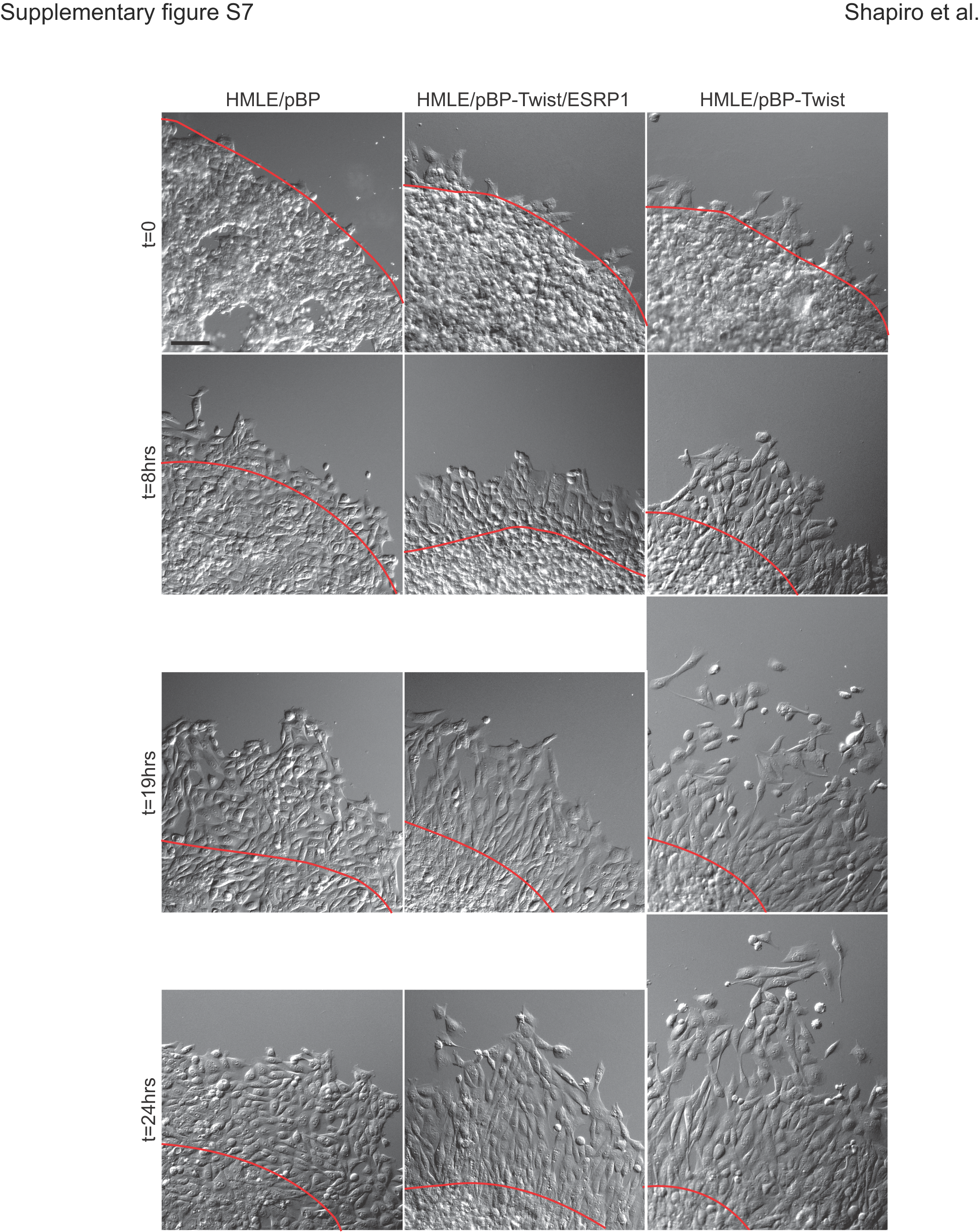

Supplement: Figure S7 — Comparison of the migration behavior of HMLE/pBP, HMLE/pBP-Twist and HMLE/pBP-Twist/ESRP1 cells. Cells were plated in a matrigel drop on top of a thin matrigel layer and allowed to migrate out of the drop for 24 hrs. Migration was followed using 10× DIC imaging at time intervals after the start of the experiment, as indicated. Red line marks the boundary of the initial matrigel drop. Scale bar, 100 µm. (TIF) [file pgen.1002218.s007.tif]

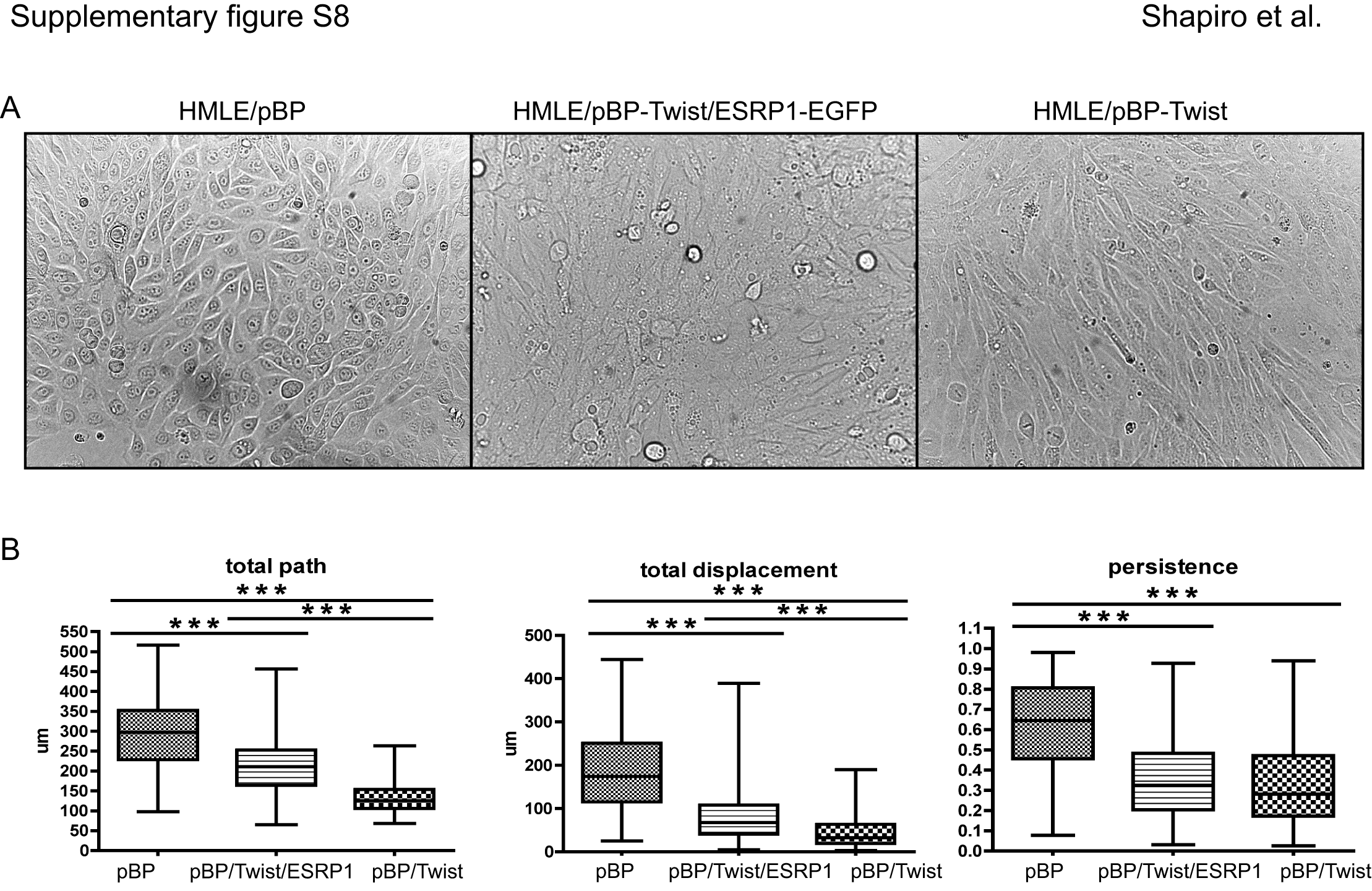

Supplement: Figure S8 — Monolayer migration assay analysis. (A) Phase contrast images of cells, as indicated, plated in a monolayer for the cell tracking experiment in Figure 6. (B) Box plots depict migration parameters inferred from live-cell imaging experiment of cells in Figure 6 and analyzed by the Imaris software. Edges of the boxes indicate 25th and 75th percentile and the whiskers 5th and 95th percentile. The line in the box indicates the median of the distribution. n = 138 cells for HMLE/pBP; n = 125 cells for HMLE/pBP-Twist; n = 113 cells for HMLE/pBP-Twist/ESRP1-EGFP. *** = p<0.001. (TIF) [file pgen.1002218.s008.tif]

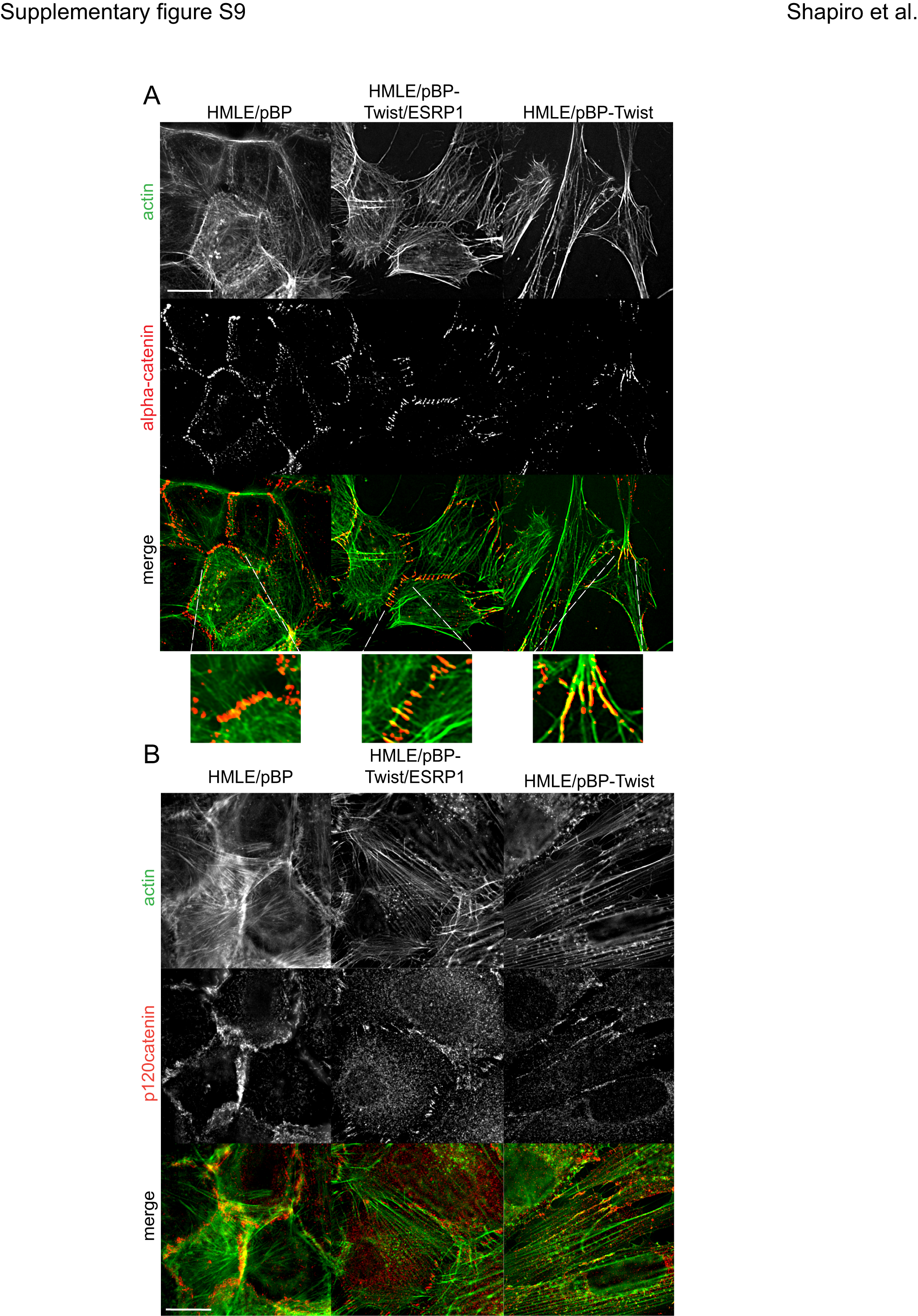

Supplement: Figure S9 — Immunofluorescence analysis of cell-cell junctions in HMLE cells. (A) and (B) Immunofluorescence of cells, as indicated, using anti-alpha-catenin (A) and p120 catenin (B) antibodies and Alexa350-phalloidin. Scale bar, 20 µm. Insets were 5× magnified. (TIF) [file pgen.1002218.s009.tif]

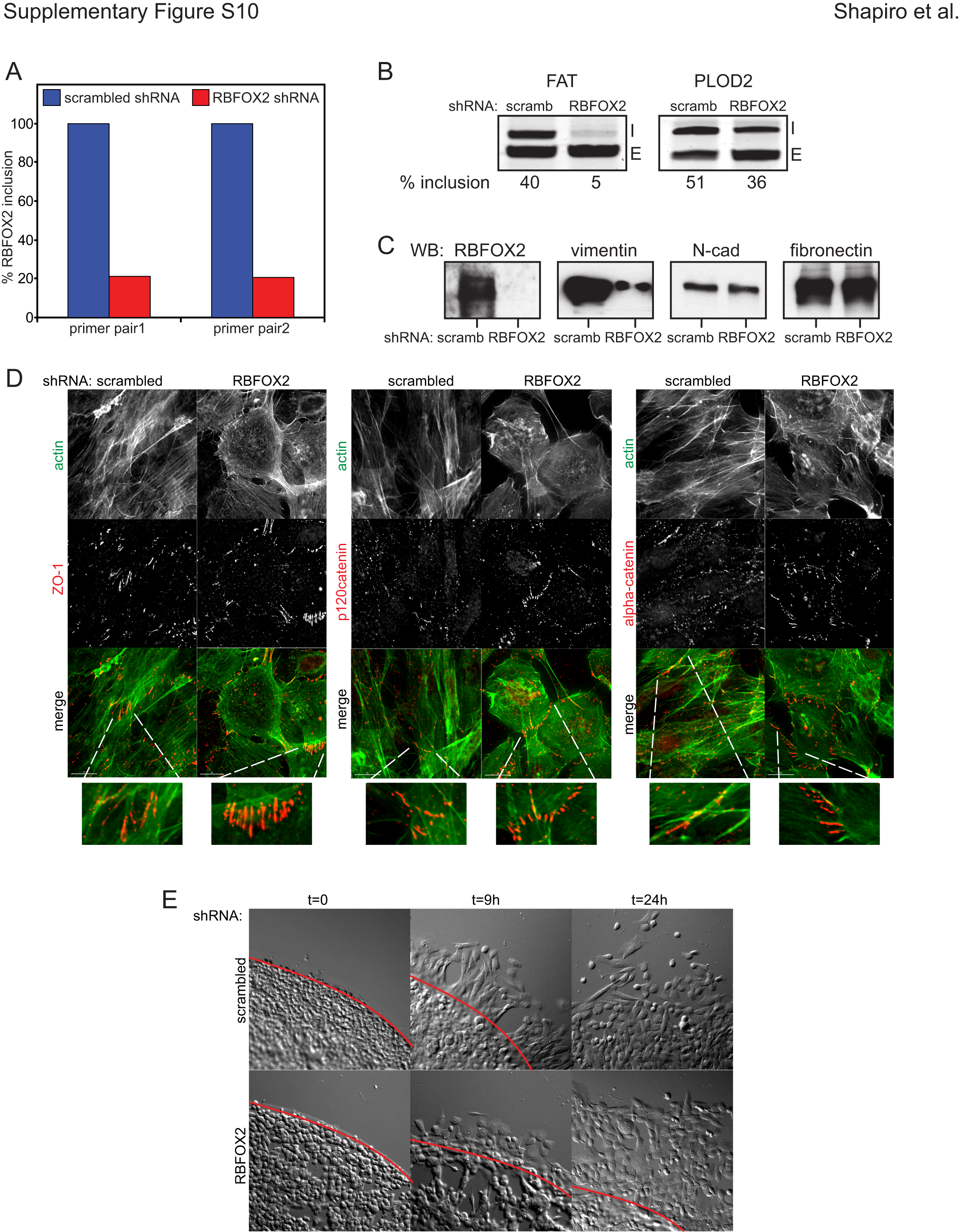

Supplement: Figure S10 — Depletion of RBFOX2 confers epithelial-like properties to mesenchymal cells. (A) qPCR analysis of RBFOX2 levels in HMLE/pBP-Twist cells expressing scrambled shRNA or RBFOX2 shRNA using two different primer pairs. (B) RT-PCR analysis of alternative exon inclusion in FAT and PLOD2 in HMLE/pBP-Twist cells expressing scrambled shRNA or RBFOX2 shRNA, as indicated. E marks excluded isoform, I marks included isoform. (C) Western blot analysis of EMT markers and RBFOX2 expression in scrambled or RBFOX2 shRNA treated cells, as indicated. (D) Immunofluorescence analysis of cell junctions using anti-ZO-1, anti-p120catenin, anti-alpha-catenin antibodies and Alexa-350 phalloidin, as indicated. Scale bar 15 µm. Insets were 5× magnified. (E) Comparison of the migration behavior of HMLE/pBP-Twist cells expressing scrambled or RBFOX2 shRNA. For a detailed description see the legend to Figure S4. (TIF) [file pgen.1002218.s010.tif]

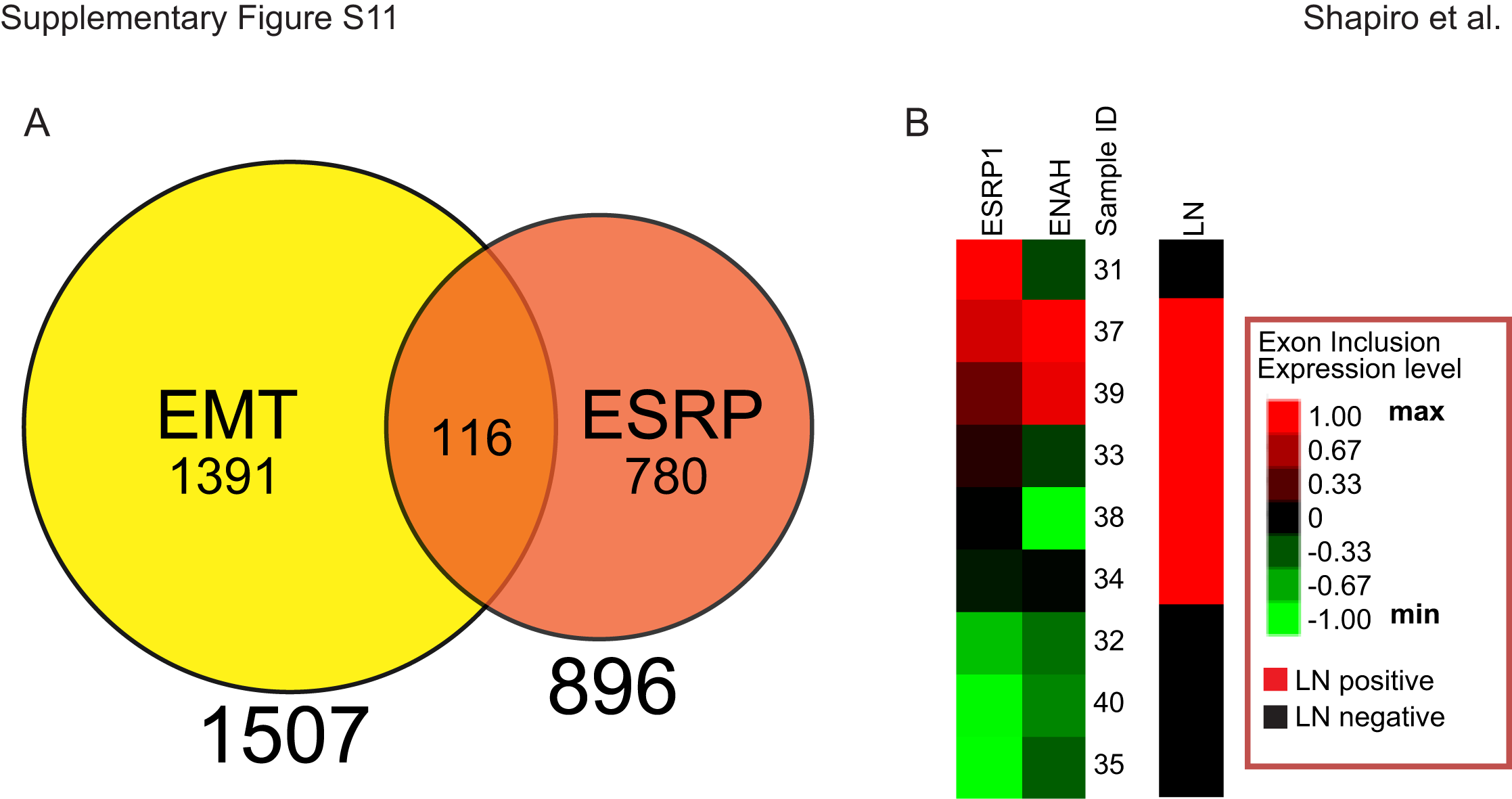

Supplement: Figure S11 — ESRP1,2 regulate a subset of EMT-dependent skipped exon events. (A) Venn diagram showing the overlap of skipped exon events reported in Warzecha et al 2009, 2010 [31], [43], and identified from our EMT RNA-seq dataset (FDR<0.05). 1391 events are unique to EMT RNA-seq dataset, 780 events are unique to the union of Warzecha et al 2009, 2010 [31], [43] datasets, 116 are common to both datasets. The numbers beneath the circles denote the number of events reported in the current study and in Warzecha et al 2009, 2010. (B) Heatmap of the ESRP expression levels and exon inclusion level of ENAH alternative exon. The expression values and exon inclusion levels are rescaled into [−1,1] and depicted as shades of red and green. Sample rows were sorted by ESRP1 expression. Sample ID is shown to the right of the heatplot. Lymphnode metastasis for corresponding samples is shown as red (LN positive) and black (LN negative) circles. (TIF) [file pgen.1002218.s011.tif]
