# Supplementary material for: An EMT–Driven Alternative Splicing Program Occurs in Human Breast Cancer and Modulates Cellular Phenotype
Source: PLoS Genet. 2011 Aug 18;7(8):e1002218. doi: 10.1371/journal.pgen.1002218 (PMC3158048; doi:10.1371/journal.pgen.1002218)
Supplement: Text S1 — Supplementary Experimental Procedures. (DOC) [file pgen.1002218.s017.doc]

**Supplementary Experimental Procedures**

**Immunofluorescence Microscopy**

Cells were plated on glass coverslips, fixed and stained as previously described [1].[Gertler et al., 1996](http://www.sciencedirect.com/science?_ob=ArticleURL&_udi=B6WW3-4V3K6DM-6&_user=501045&_coverDate=12%2F09%2F2008&_rdoc=1&_fmt=high&_orig=search&_sort=d&_docanchor=&view=c&_acct=C000022659&_version=1&_urlVersion=0&_userid=501045&md5=1687fd5546bb1dddd9555e1ce69bc1f2#bbib21) F.B. Gertler, K. Niebuhr, M. Reinhard, J. Wehland and P. Soriano, Mena, a relative of VASP and *Drosophila* Enabled, is implicated in the control of microfilament dynamics, *Cell* **87** (1996), pp. 227–239. [**Article**](http://www.sciencedirect.com/science?_ob=ArticleURL&_udi=B6WSN-41BD859-C&_user=501045&_coverDate=10%2F18%2F1996&_fmt=full&_orig=search&_cdi=7051&view=c&_acct=C000022659&_version=1&_urlVersion=0&_userid=501045&md5=5ea515adf74711483f92ded1294dda73&ref=full) || [View Record in Scopus](http://www.sciencedirect.com/science?_ob=RedirectURL&_method=outwardLink&_partnerName=655&_originPage=article&_zone=art_page&_targetURL=http%3A%2F%2Fwww.scopus.com%2Finward%2Frecord.url%3Feid%3D2-s2.0-0030592559%26partnerID%3D10%26rel%3DR3.0.0%26md5%3Df0761d77959984799a3d107f8129d242&_acct=C000022659&_version=1&_userid=501045&md5=8c0a4b542cfb17beae5094f0637c0335) | Cited By in Scopus (396) AlexaFluor405 phalloidin (Molecular Probes) was used at 1:100. Cells were imaged using a Deltavision-OMX or a Deltavision microscope (Applied Precision, Olympus IX71, 60X/1.4NA Plan Apo objective) and processed using a Softworx software (SGI, Mountain View, CA).

**Plasmids, virus production and infection of target cells**

The pMSCV-ESRP1-GFP construct was generated by replacing the Mena cDNA of pMSCV-Mena-GFP [2] with the hESRP1 cDNA (Open Biosystems, clone LIFESEQ3617421). Retroviral packaging, infection, and fluorescence-activated cell sorting (FACS) were performed as previously described [3]. Short hairpin RNA (shRNA) for the knockdown of RBFOX2 was described previously [4] as pBlsH1Fox-2. Hairpin was subcloned into pLKO.1 vector for lentivirus production and infection as described previously [5].[Bear et al., 2000](http://www.sciencedirect.com/science?_ob=ArticleURL&_udi=B6WW3-4V3K6DM-6&_user=501045&_coverDate=12%2F09%2F2008&_rdoc=1&_fmt=high&_orig=search&_sort=d&_docanchor=&view=c&_acct=C000022659&_version=1&_urlVersion=0&_userid=501045&md5=1687fd5546bb1dddd9555e1ce69bc1f2#bbib5) J.E. Bear, J.J. Loureiro, I. Libova, R. Fassler, J. Wehland and F.B. Gertler, Negative regulation of fibroblast motility by Ena/VASP proteins, *Cell* **101** (2000), pp. 717–728. [**Article**](http://www.sciencedirect.com/science?_ob=ArticleURL&_udi=B6WSN-4194S9G-6&_user=501045&_coverDate=06%2F23%2F2000&_fmt=full&_orig=search&_cdi=7051&view=c&_acct=C000022659&_version=1&_urlVersion=0&_userid=501045&md5=829fb3a34011ab6be98f548f1687adcb&ref=full) | [
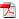
PDF (560 K)](http://www.sciencedirect.com/science?_ob=MiamiImageURL&_imagekey=B6WSN-4194S9G-6-K&_cdi=7051&_user=501045&_pii=S0092867400808843&_check=y&_orig=search&_coverDate=06%2F23%2F2000&view=c&wchp=dGLbVzb-zSkzS&md5=334db0af346e424e4905df3797ae434f&ie=/sdarticle.pdf) | [View Record in Scopus](http://www.sciencedirect.com/science?_ob=RedirectURL&_method=outwardLink&_partnerName=655&_originPage=article&_zone=art_page&_targetURL=http%3A%2F%2Fwww.scopus.com%2Finward%2Frecord.url%3Feid%3D2-s2.0-0034705317%26partnerID%3D10%26rel%3DR3.0.0%26md5%3D9445efb950cbb1d75470e6fb7c8187df&_acct=C000022659&_version=1&_userid=501045&md5=b18ae89c0955a4a566b592d83565e7a5) | [Cited By in Scopus (219)](http://www.sciencedirect.com/science?_ob=RedirectURL&_method=outwardLink&_partnerName=656&_originPage=article&_zone=art_page&_targetURL=http%3A%2F%2Fwww.scopus.com%2Finward%2Fcitedby.url%3Feid%3D2-s2.0-0034705317%26partnerID%3D10%26rel%3DR3.0.0%26md5%3D9445efb950cbb1d75470e6fb7c8187df&_acct=C000022659&_version=1&_userid=501045&md5=12ee712d2dbcacf5e05cb01e96f5b293){Stern, 2008 #5}{Stern, 2008 #5}

**qPCR analysis quantification**

GAPDH mRNA was used to normalize RNA inputs. 2 pairs of primers were used for each alternative splicing event. One pair of primers was complimentary to a region outside alternative exon (out), the other pair (int) had one primer internal to alternative exon and the other primer flanking alternative exon. All quantitations were normalized to an endogenous control GAPDH. The relative inclusion value for each target splicing event was expressed as 2^-(Ct(int)-Ct(out))^ (Ct(int) and Ct(out) are the mean threshold cycle differences after normalizing to GAPDH). To eliminate primer efficiency issue, all relative inclusion values for IDC samples were normalized, i.e. depicted as a fold difference to the average FA inclusion value based on the analysis of six fibroadeoma samples.

**Primers used for qPCR analysis of cell line cDNA and FNA samples cDNA:**

| Gene name | Alternative exon  Forward primer 5’-3’ | Alternative exon  Reverse primer  5’-3’ | Outside region  Forward primer  5’-3’ | Outside region  Reverse primer  5’-3’ |
| --- | --- | --- | --- | --- |
| GAPDH | CATGAGAAGTATGACAACAGCCT | AGTCCTTCCACGATACCAAAGT | N/A | N/A |
| ENAH | CAACAAGAAAACCTTGGGAAA | GGACCTGTTGTCAAAAACAATCT | GAACAAAAAGAGGACAAAGGTGA | TGCCATTCATTGTATTTGTTCTTT |
| SLC37A2 | GGTCCTAACCCACCAGTGAT | ACTGGGACCCTCCATGCT | GGGCTGAGTTGTGTCTCCAT | GAGAGATGCCCATTTTCCAG |
| MBNL1 | CTCAGTCGGCTGTCAAATCA | AGAGCAGGCCTCTTTGGTAA | ttcatccacccccacattta | ttggctagttgcatttgctg |
| FLNB | TGTGATCTATGTGCGCTTCG | CATTTACCGGTGCCTCCTC | ATCGCCTCCACTGTGAAAAC | AGTGCCATCTGGGGTCAG |
| ARHGEF11 | TGGCATGCTGACATAAAAGC | GGTTGTCCCTGCACTACCAG | TGACAGAAGGTGTGGGTGTC | AACCTGCGACATCTGATCCT |
| MLPH | GATGGCCTCCCACCATTC | CAGGTAGGTCAGCAGGCATT | AGGAAGCTGGAGGAGCTGAC | CCCAACTGATTTGTCCCTGT |
| KIF13A | CAGGGTTATGTGCCTGAGGT | CAAGCCCCTAATGCCTGTAA | AGAAGGGACCACCATGTCAG | CTCACGGGTCTTGGAGAAAG |
| PLOD2 | GCAGTGGATAATAGCCTTCCA | GACTCCCCTACTCCGGAAAC | CTAGCATTTCGGCAAAGAGC | TGTACTTAATTAAAGGAAAGACACTCC |
| PLEKHA1 | AAGGCTGTCGAACCCTTGTA | GAGGCTGTGGAATGTGAGGT | GTCAAGCCAGGGAACTTCAA | TTTCCTGAGGGCCATTTTTA |
| CLSTN1 | CACCTTCTTATCCGCGAGTT | AACTGAGCCTGTGACTGTGG | GAGCGGGTAATCCTCAGTCA | AATGGCACCACTACGTCCTC |

Primers used for the semi-quantitative RT-PCR Skipped Exon events analysis:

| Gene name | Forward primer 5’- 3’ | Reverse primer 5’-3’ |
| --- | --- | --- |
| SLC37A2 | ctagcctgcttgctcctttg | Tggaagtttccattgtcttgc |
| CUGBP1 | AGAGTTCCCGCAAGTCCTTT | TCAAAGTTCCCTGTGTTGTGA |
| ENAH | GGTGAAGATTCAGAGCCTGTAACTTC | CACTGGGCTGTGATAAGGGTG |
| MBNL1 | CATTTGCAAGCCAAGATCAA | TGGGGGAAGTACAGCTTGAG |
| FLNB | TCCTAACAGCCCCTTCACTG | TTCCTGACAGCAAACGGAAT |
| KIF13A | GTTCTCCAAGCTGGCATTGT | GGCCTCTTCTAAGCCAGGAG |
| MICAL3 | GGTCAGCTTGGCATTCAGTT | AGCTGAGCTTCTCCGAGGAC |
| MICAL3 | GCTGCCTCCCCTTCTATCTC | acgaggaggaggaagagTCC |
| PACSIN3 | CTTAGCTGCTGCTGGCTTCT | ACCTCACCCAAAGCCTCACT |
| EPB41L1 | AGCTGGTGTGGACAGAGGAG | CGGCCTCACTGTAGTCCTTC |
| ARHGEF11 | TGGCATGCTGACATAAAAGC | AGAGGCAGCAGGAGGTTACA |
| PLEKHA1 | GCCCTGAAGAGATGCACAGT | GAGGCTGTGGAATGTGAGGT |
| CLSTN1 | TCGGAAAAACTGGGTCATGT | AATGGCACCACTACGTCCTC |
| PLOD2 | CAAAAATCTGCCAGAGGTCA | GATATGGCTCTTTGCCGAAA |
| MLPH | AGTACTTGGCCGATGTGGAC | CTCAGGGCCTCCTCCTCTAC |
| SNX14 | GCAACATAGCTCCCTCCATT | AATCACCAACACGCAATTCA |
| PPFIBP1 | CGGCACTCGAAAAGTCAGAT | CCAGCCAGATCTAGGTGCTC |
| FAT | TGTATGTCCGGCAGAGGAAC | GGAAAGCCTGTCTGAAGTGC |
| NEK1 | TAATCTGTTGGCGCTCATTG | AAACGGGAAGCTATGCAGAA |
| NFYA | GGATCTCCAGAGTGGACAGG | TCCACTGACCTGCACCATTA |
| ROBO1 | TTCGCCTCCTCTCTGGTAAG | ACCCTGTGTCACCTGAGGAC |
| DTNB | TGTATGTGGTGACCCTGTGG | AGGAAGGATGAACTGGAGCA |
| STX2 | TCCAAGGATCACAAGCAAAA | ATCAGAGCAAGGCAAGAAGG |
| CSNK1G3 | GTTCAAATGCACCCATCACA | CCCCAGGATCTGTCTGTGTC |
| TEAD1 | CTTGCCAGAAGGAAATCTCG | CAGCCCCAGCTTGTTATGAA |
| VDP | GAAAATGCCACCCAGAAAGA | TGCAATGGGACAATTGCTTA |
| ATP5C1 | GCCAAGCTGTCATCACAAAA | GGACAAAGGCAGCAGTAAGC |
| TSC2 | CGGTCCAATGTCCTCTTGTC | CACTGGTGAGGGACGTCTG |
| ASXL1 | GCCTCGAGTTGTCCTGACTC | TCTGTTGCGCTTCATTTGAC |
| APLP2 | CATGTCAGACAAGGAAATTACTCA | ATCATTGGTTGGCAGAGGAG |
| CENTD3 | TCCTCGTACACAGGCTCCTC | TATGCCTTTGCTGCCTATCC |

**Primers used for semi-quantitative RT-PCR** Mutually Exclusive Exon analysis:

| Gene name | Forward flanking primer | Reverse internal primer1 | Reverse internal primer 2 |
| --- | --- | --- | --- |
| FGFR2 | CAGGTAGTCTGGGGAAGCTG | GCAGAAGTGCTGGCTCTGTT | CACCACGGACAAAGAGATTG |
| FGFR1 | GCCCCTGTGCAATAGATGAT | AATGTGACAGAGGCCCAGAG | ACCACCGACAAAGAGATGGA |
| DOCK9 | TCAGGCAAACCTCAGTAGCA | ACATTGCCTGTTTCCCGTAA | TCAAGTGTGCTTGGAATTTCTG |
| Septin-2 | CAAGGCGAAGATTCTCATTACC | GCTGCCAAATGAGTTTTGGT | ccttggacaagaccaaagtca |

### Mapping of sequencing reads.

Sequencing reads were preprocessed by the Illumina/Solexa Pipeline. MAQ [6] was used for mapping reads to the hg18 human genome and junction database. The Acembly gene annotation [7] was used to define exon boundaries and splicing junctions. A junction database was generated by concatenating exonic sequences at junctions. For 39 nucleotide (nt) reads, 38nt from both upstream and downstream exons of a junction were concatenated to represent the junctional sequence. The reference transcriptome on which reads were mapped was the union of the junction database and the genome. The sequence of mTwist cDNA was included in the reference to account for the expression of mTwist during induction of EMT. Only uniquely mapped reads with less than 3nt mismatches were retained. In addition, to ensure the fidelity of mapping to junctions, a junctional read was kept for subsequent analysis only when both exons flanking the junction were covered by at least 4nt on the reads. Uniquely mappable positions were found by simulating all reads from both strands of the genomic and the junctional sequences and filtering for positions with unique sequences. These uniquely mappable positions were used in subsequent analysis as the effective lengths of exons. To assess a potential contamination from rRNA, we attempted mapping of all reads onto an rRNA reference constructed from human rRNA sequences downloaded from Silva databases (<http://www.arb-silva.de/>).

### Inference of gene expression levels.

Inference of Gene expression levels was guided by pre-defined transcript annotation from RefGene [8]. To eliminate biases in estimating gene expression due to alternative splicing resulting in some regions of the transcripts differentially present between samples, only constitutively expressed regions, i.e., regions expressed in all annotated transcripts, of a gene were considered. Because the protocol used for mRNA-Seq in this study did not provide strand information of the original template, ambiguous regions where there were annotated transcriptions from both strands on the genome were ignored. Noise for expression analysis was modeled as a Poisson random variable parameterized by reads mapping to non-exonic regions of the genome. Let P_g_ be the probability for a read to land on exons of gene g and l_g_ be the length of the exons of gene g, l_e_ be the sum of the length of all exonic regions in the genome. P_g_= l_g_/l_e_. Let r_e0_ be the number of noisy reads distributed onto exonic regions in the genome, d_ne_ be the density of reads aligning in non-exonic regions of the genome. r_e0_ is estimated from non-exonic read density, i.e., r_e0_=d_ne_ x l_e_. Random variable X_g_ is the number of reads aligned on gene g under the null model and X_g_~Poisson(λ=r_e0_p_g_=l_g_d_ne_). The p-value for expression of a gene with x reads aligned can thus be derived as P(X_g_≥x,,λ=r_e0_p_g_). Benjamini-Hochberg (B-H) false discovery rate (FDR) procedure [9] was used to get FDR for expression (FDR_exp_). TMM normalization [10] were used to find a scaling normalization factor for normalizing expression in mesenchymal sample using epithelial sample as the reference. Gene expression values were expressed in Reads Per Kilobase of Exon Model Per Million Mapped Reads (RPKM) which normalizes read counts to length of exons and total reads from the sample mapped to the reference [11]. We added the poisson noise λ=r_e0_p_g_ to each gene’s read counts such that RPKM from genes with no reads aligned can be log-transformed. The RPKM values were normalized using the TMM normalization constant. Let E be pre-EMT sample, M be post-EMT sample. We used two criteria (DE1 and DE2) for differential expression. For DE1, We used the Audic-Claverie statistics [12] requiring B-H FDR for differential expression (FDR_de_) <0.05. For DE2: we applied an arbitrary threshold of 3 fold. Differentially expressed (DE) genes were classified into two classes. Class 1 DE genes were those satisfying DE1 criterion but not DE2 criterion. Class 1 DE genes were labeled as either “Up” or “Down”, if RPKM(M)>RPKM(E) and RPKM(E)>RPKM(M), respectively. Class 2 DE genes were those satisfying both DE 1 and DE2 criteria. These genes were labeled as either “Up3x” or “Down3x”, for 3 fold up or 3 fold down from epithelial to mesenchymal cells, respectively. Genes were called ubiquitously expressed or not changed if FDR_exp_ in both samples <0.05 and did not pass DE1 criterion. Genes were labeled as “not expressed” if FDR_exp_>=0.05.

### Inference of alternative mRNA processing events and alternative transcription initiation.

Inference of alternative mRNA processing events and alternative transcription initiation was guided by transcript annotation information from AceView [7]. A splice graph was constructed from all transcripts annotated for a gene such that exons were represented by nodes and edges were formed by connecting exons when there is a junction between them. The splice graph was traversed to identify splicing events depicted in Figure 2. To quantify splicing, we used a measure called “Percent spliced-in” or Psi (Ψ) [13]. Ψ was calculated by dividing the inclusion read density by the sum of the inclusion and exclusion read densities. Inclusion and exclusion isoforms were defined differently for different AS events and are illustrated in Figure 2. Calculation of Ψ value for each event is similar to Wang *et al*., 2008 [13], with slight changes to the filters, briefly: inclusion reads (NI) are the reads that are mapped to the inclusion junction(s) or the inclusion-specific (cassette) exon body. Exclusion reads (NE) are the reads that are mapped to the exclusion junction(s) or the exclusion-specific exon body (if applicable). In SE, RI, 5’AltSS, 3’AltSS, NE+ is the sum of exclusion reads plus the reads that are mapped to flanking exons; otherwise NE+ is just exclusion reads. Fisher’s exact test was performed on a 2x2 table using NI and NE+ from the two samples. An event is detectable if inclusion pos (IP) >=1 and exclusion pos (EP) >=1. At least one isoform of an event is detected if the event is detectable and that inclusion reads (NI) + exclusion reads (NE) in both samples >=1 and that NI and NE in the pooled sample >=10. Both isoforms of an event are detected if the event is detected and both NI >=1 and NE >=1 in the pooled sample. Correction for multiple testings for the Fisher’s exact test was performed by B-H FDR procedures on “both isoform detected” set. The “significant AS events” (sigset) were selected from the set of “both isoform detected” events where FDR<0.05 and |ΔΨ| >= 0.1. For gene ontology (GO) enrichment analysis, we defined a set of background events with enough read coverage to detect significant events as the “powerset”. The minimal inclusion-exclusion reads and minimal NI, NE+ reads required to give power to detect was decided by finding the following bounds within the sigset: Let NI be inclusion reads, NE be exclusion reads and NEp be NE+ reads.

To account for Ψ calculation as a function of NI and NE, we defined two bounds:

B1=min over events i and samples j [ NI(i,j) + NE(i,j) ]

B2=min over events i ( sum over samples j [ NI(i,j) + NE(i,j) ] )

To account for Fisher exact test as a function of NI and NE+, we define B3 and B4 statistics:

B3=min over events i and samples j [ NI(i,j) + NEp(i,j) ]

B4=min over events i (sum over samples j [ NI(i,j) + NEp(i,j) ])

The set with the power to detect alternative splicing (powerset) was selected from the “both isoform detected” subset of known events where for each event i:

min over samples j [ NI(i,j) + NE(i,j) ] >= B1

sum over samples j [ NI(i,j) + NE(i,j) ] >= B2

min over samples j [ NI(i,j) + NEp(i,j) ] >= B3

sum over samples j [ NI(i,j) + NEp(i,j) ] >= B4

In order to ensure that the sigset and powerset have similar distribution of B4 statistics, we iteratively scale up B4 and other bounds in proportion, until the median of (sum over samples j [ NI(i,j) + NEp(i,j) ]) of the powerset is equal or slightly higher than that of the sigset. The background set was defined as the union of the powerset and the sigset..

### Motif enrichment analysis and prediction of mRNA processing factors operating in EMT

Significant SE events were divided into two sets. Upregulated sets are those with FDR of alternative splicing FDR(AS)<0.05 and ΔΨ>0.1. Downregulated sets are those with FDR (AS)<0.05 and ΔΨ<-0.1. These two sets were subjected to motif enrichment analysis separately. 250bp regions of introns flanking epithelial- or mesenchymal-specifically spliced exons and the upstream and downstream exons were collected for motif enrichment analysis. Sequences were divided into equally sized (100 sequences/bin) bins according to composition of G and C nucleotides (%GC). A separate background pentamer (5mer)-generating first-order Markov model (1MM) was built from mononucleotide and bi-nucleotide frequencies of the sequences in each bin. Background probability of a 5mer was calculated per bin and averaged to get the overall background probability. The actual frequency of a 5mer was obtained by counting its occurrences in all foreground sequences. p-value of a 5mer was calculated by a binomial complementary cumulative density function (ccdf) of its observed frequency over the background probability distribution generated by the 1MM. To find motifs enriched in EMT-regulated exons relative to the non-regulated exons, another analysis based on hypergeometric enrichment of 5mers was performed. The number of occurrences of 5mers was counted from foreground, i.e., the significant set of events (FDR(AS)<0.05 and ΔΨ>0.1 or ΔΨ<-0.1), and the corresponding background (union of the significant set with the powerset). To account for CG%, the foreground was first binned into CG% bins. The background sequences were similarly binned. The background sequences were randomly sampled per bin proportional to the bin sizes in the foreground. A hypergeometric enrichment p-value was calculated using the foreground and background frequencies. Correction for multiple testing for both motif analyses was done following B-H FDR procedure.

Expression of RNA binding proteins and splicing factors was explored by finding annotated RNA binding proteins and splicing factors (according to gene ontology annotation and a list of known splicing factors) from the expression data. To overlap EMT events with published CLIP-seq data, binding clusters or binding sites from published CLIP-seq experiments of various splicing factors were compared against the sigsets and powersets of the different EMT events. PTB data was obtained from bed-formatted interval files from GEO database entry [14]. SFRS1 CLIP-data were bed files obtained from Sanford Lab website [15]. FOX2 data was downloaded from UCSC genome browser [16]. CLIP-seq clusters from these studies were overlapped with the differentially regulated events in this current study to get the fraction of events in the sigset or the powerset with or without overlaps with CLIP-seq clusters. ESRP1,2 RNAi-seq and over-expression-sequencing data was kindly provided by R. Carstens (Personal Communication;[17]). EMT and ESRP1,2 data were overlapped by matching inclusion exon coordinates. The p-values for enrichment or depletion of overlaps were calculated by fisher exact test.

### Gene ontology (GO) enrichment analysis

Genes were mapped to GO_BP_FAT (Biological Processes) and KEGG pathway annotations using DAVID tool [18,19]. Statistical significance of term enrichment was derived from hypergeometric enrichment p-value of foreground annotation overlap over background annotation overlap for each term. Only terms with >=20 and <=100 genes annotated in the background were tested. In addition, terms with <10 genes annotated in the foreground set were discarded. For expression GO enrichment analysis, up-regulated genes were selected as genes with FDR(exp)<0.05 in mesenchymal sample, FDR(DE)<0.05 and RPKM(M)/RPKM(E)>=3 (Class 2 DE genes, upregulated subset). Down-regulated genes were selected as genes with FDR(exp)<0.05 in epithelial sample, FDR(DE)<0.05 and RPKM(E)/RPKM(M)>=3 (Class 2 DE genes, downregulated subset). These were used as foreground lists. To account for the fact that power of detecting differential expression increases with coverage, background list was composed of the union of the foreground set and a subset of all detectable genes (genes with strand-unambiguous and uniquely mappable positions in constitutive regions) where total number of reads mapped to the analyzed regions of that gene in two samples is bigger than or equal to the lower bound of that in the foreground set. For alternative splicing GO enrichment analysis, significant events (FDR<0.05, |ΔΨ|>0.1) from all event types were selected, collapsed into unique gene names. The background set consisted of union of the significant set and power set collapsed into unique gene names. B-H procedure was used to account for the false discovery rate associated with multiple comparisons.

**NCI-60 breast cancer cell lines exon array and EMT RNA-seq comparison**

Cancer cell line exon array data were obtained from GEO database record GSE16732 [20]. The RMA-processed matrix (in log2) was used for the analysis. Probe sets were remapped to AceView/acembly exons requiring 10bp overlap. Probesets targeting the same exon were summarized into a single exon value by taking the median. Exons with exon value < log2(10) were discarded. SE Events from AceView were used to combine exon values into event values as Inclusion probe ratio (IPR). IPR was defined per sample and event as IPR(event,sample)=CX(event,sample)-[UFX(event,sample)+DFX(event,sample)]/2 where CX(event,sample), UFX(event,sample), DFX(event,sample) are the exon values of the cassette exon, upstream flanking exon and the downstream flanking, respectively. The set of IPR values of the events that were detected in EMT RNA-seq (FDR<0.05, |ΔΨ|>0.1) as well as in the exon array (307 events) were used to cluster the cancer cell lines. These values were row-centered by median and row-normalized before hierarchical clustering using Pearson correlation and average linkage. As a control, the powerset + foreground set of events (8839) were also overlapped with the array data and their IPR values were clustered the same way. To assess the quality of a clustering classification, we derived a simple metric that assessed how well the clustering of the selected splicing events on the cancer cell lines IPR values separated the cancer cell lines into luminal and basal B subtypes [21] by counting the number of outliers. First, the clustering tree was divided into two subtrees rooted by the two children of the root. Each subtree was then treated as a group where the leaves of the subtree are the members of that group. For each group (subtree), we counted the number of basal B cell lines and luminal cell lines. Cell lines of the minority cell type were treated as outliers. The total number of outliers was the sum of outliers in the two groups. To test the significance of the clustering classification, we performed a randomization-clustering procedure. Random sets of 307 events (same size as the foreground set) were chosen from the background set and subjected to clustering using the same metrics as we did with the foreground set. The randomization-clustering procedure was repeated 10000 times. The p-value was derived as the number of random sets with fewer or the same number of total outliers as/than resulted from clustering of the foreground set divided by the total number of random sets tested (i.e., 10000).

To find a subset of “core” EMT splicing events that separates the luminal and basal B groups, the following procedures were undertaken: cell lines were grouped into Luminal and Basal B groups according to [21] and listed below. Inclusion probe ratio (IPR) per sample group per event was defined using the mean exon values of that group by IPR(event,group)=CXmean(event,group) – [UFXmean(event,group)+DFXmean(event,group) ]/2 where CXmean(event,group), UFXmean(event,group) and DFXmean(event,group) denote the group mean exon values for cassette exon, upstream flanking exon and downstream flanking exon, respectively. The variance was estimated by IPRvar=CXvar(group)+ [UFXvar(group)+DFXvar(group)]/4. Splicing changes ("differential inclusion ratio" - DIR) were inferred by subtracting IPR(Luminal) from IPR(BasalB). A value >0 indicates more inclusion isoform in basal cells. A value <0 indicates more inclusion isoform in luminal cells. The significance of the change detected by array were assessed by a Welch t-test on the IPR and the standard error of the mean (SEM) derived from IPRvar of the basal and luminal samples. Array-detected events with FDR<0.25 were selected for subsequent analysis. These events were compared to the set of significant EMT Skipped Exon (SE) events (FDR<0.05, |ΔΨ|>0.1, 0.2, or 0.3). Of 481 significant RNA-seq SE events, 268 were detected by array above the probe detection threshold, of which, 24 were called significantly changed in the array (array FDR<0.25, EMT RNA-Seq FDR<0.05, |ΔΨ|>0.1). A coherent event was defined as an event called significant in both the NCI-60 cancer cell line exon array dataset [20] and EMT RNA-Seq data and had the same direction of change in EMT as in comparison of luminal to basal B cell lines. Clustering analysis of breast cancer cell lines from the NCI-60 panel was performed using the 19 coherent events from 24 events called significantly changed in both exon array data and RNA-Seq data. The IPR values were used event (row)-centered by median and event-normalized such that sum of squares per event equals 1. Hierarchical clustering using Pearson correlation and average linkage was performed on the transformed data.

**Breast cancer cell lines from the NCI-60 panel [20] used for analysis:**

**Luminal:**
GSM419256       BT474
GSM419257       BT483

GSM419259       CAMA-1
GSM419264       MCF7
GSM419265       MDA-MB-134VI

GSM419267       MDA-MB-175VIII
GSM419270       MDA-MB-361
GSM419271       MDA-MB-415

GSM419274       MDA-MB-453
GSM419279       SKBR-3
GSM419285       SUM185
GSM419289       SUM44
GSM419290       SUM52
GSM419291       T47D
GSM419292       UACC812

GSM419294       ZR751
GSM419295       ZR7530

**Basal B:**
GSM419258       BT549

GSM419263       Hs578T

GSM419266       MDA-MB-157

GSM419268       MDA-MB-231

GSM419273       MDA-MB-436

GSM419282       SUM1315
GSM419283       SUM149
GSM419284       SUM159

**Hierarchical clustering of FNA samples**

For each splicing event, qRT primers targeting cassette exon and an independent primer pair targeting a constitutive region of the host gene were used to measure relative inclusion levels of the cassette exon across samples. The exon inclusion values were calculated as Ct value of the reaction with the constitutive primer pair minus the Ct value of the reaction with the cassette exon primer pair. Values from FNA samples for each event were rescaled into [-1,1] scale by 2(x-min)/(max-min)-1. Hierarchical clustering of FNA samples were done on Ratio to Average (RA) values (fold change to average inclusion ratios in fibroadenoma samples) using Biopython ([http://biopython.org](http://biopython.org/)) Cluster3 module (Pearson correlation, average linkage) [22]. Cluster tree and heatmap was visualized in JavaTreeView [23]. Unsupervised hierarchical clustering (Pearson correlation, average linkage) were performed on both the event vectors (columns) and the sample vectors (rows) using Cluster3[24]. The significance of the separation of the epithelial markers and mesenchymal markers were assessed by shuffling the columns and repeating clustering on the randomized matrices. The randomization-clustering procedures were repeated 10,000 times. The furthest distance of the event clustering tree was defined as the distance between the top two subtrees. The p-value is defined as the fraction of randomized matrices achieving at least as far max distance as the data matrix (i.e., the Pearson distance between the epithelial events and the mesenchymal events). In addition, bootstrapping analysis was performed on the data matrix to assess the reliability of the event clustering tree using pvclust R package [25]. ESRP1 expression values (Ct of control GAPDH reaction minus Ct of ESRP reaction) were rescaled to [-1,1] scale by 2(x-min)/(max-min)-1. The Mena/11a exon inclusion vectors from these samples were similarly rescaled as above. The ESRP- Mena/11a heatmap was sorted with ascending values on the ESRP column.

**Method for assessing dependency between gene expression and alternative splicing**

To assess dependency of gene expression changes and alternative splicing regulation, we compared the cumulative distribution of log expression changes during EMT in the set of genes differentially spliced during EMT (foreground) and a background set of genes which are not differentially spliced during EMT. Kolmogorov-Smirnov (KS) test was performed on the CDF curves to estimate the p-value of the distribution differences.

**Method for estimating overlap of EMT and Warzecha et al. ESRP1 KD and OE data [26], 15]**

To ensure fair comparison of the data, we uses the set of gene we have confident to believe to be alternative spliced during EMT by an FDR<0.05. We compared this set of 1507 events with those defined in Warzecha et al ESRP1 over-expression and knockdown experiments by matching the genomic coordinates of the cassette exons.

**Text S1 References**

1. Gertler FB, Niebuhr K, Reinhard M, Wehland J, Soriano P (1996) Mena, a relative of VASP and Drosophila Enabled, is implicated in the control of microfilament dynamics. Cell 87: 227-239.

2. Philippar U, Roussos ET, Oser M, Yamaguchi H, Kim HD, et al. (2008) A Mena invasion isoform potentiates EGF-induced carcinoma cell invasion and metastasis. Dev Cell 15: 813-828.

3. Bear JE, Loureiro JJ, Libova I, Fassler R, Wehland J, et al. (2000) Negative regulation of fibroblast motility by Ena/VASP proteins. Cell 101: 717-728.

4. Tang ZZ, Zheng S, Nikolic J, Black DL (2009) Developmental control of CaV1.2 L-type calcium channel splicing by Fox proteins. Mol Cell Biol 29: 4757-4765.

5. Stern P, Astrof S, Erkeland SJ, Schustak J, Sharp PA, et al. (2008) A system for Cre-regulated RNA interference in vivo. Proc Natl Acad Sci U S A 105: 13895-13900.

6. Li H, Ruan J, Durbin R (2008) Mapping short DNA sequencing reads and calling variants using mapping quality scores. Genome research 18: 1851.

7. Thierry-Mieg D, Thierry-Mieg J (2006) AceView: a comprehensive cDNA-supported gene and transcripts annotation. Genome Biology 7: S12.

8. Pruitt K, Tatusova T, Maglott D (2006) NCBI reference sequences (RefSeq): a curated non-redundant sequence database of genomes, transcripts and proteins. Nucleic acids research.

9. Benjamini Y, Hochberg Y (1995) Controlling the false discovery rate: a practical and powerful approach to multiple testing. Journal of the Royal Statistical Society Series B (Methodological): 289-300.

10. Robinson MD, Oshlack A A scaling normalization method for differential expression analysis of RNA-seq data. Genome Biol 11: R25.

11. Mortazavi A, Williams B, McCue K, Schaeffer L, Wold B (2008) Mapping and quantifying mammalian transcriptomes by RNA-Seq. Nature methods 5: 621-628.

12. Audic S, Claverie J (1997) The significance of digital gene expression profiles. Genome research 7: 986.

13. Wang E, Sandberg R, Luo S, Khrebtukova I, Zhang L, et al. (2008) Alternative isoform regulation in human tissue transcriptomes. Nature 456: 470-476.

14. Xue Y, Zhou Y, Wu T, Zhu T, Ji X, et al. (2009) Genome-wide Analysis of PTB-RNA Interactions Reveals a Strategy Used by the General Splicing Repressor to Modulate Exon Inclusion or Skipping. Molecular cell 36: 996-1006.

15. Sanford J, Wang X, Mort M, VanDuyn N, Cooper D, et al. (2009) Splicing factor SFRS1 recognizes a functionally diverse landscape of RNA transcripts. Genome research 19: 381.

16. Yeo G, Coufal N, Liang T, Peng G, Fu X, et al. (2009) An RNA code for the FOX2 splicing regulator revealed by mapping RNA-protein interactions in stem cells. Nature structural & molecular biology 16: 130.

17. Warzecha CC, Jiang P, Amirikian K, Dittmar KA, Lu H, et al. An ESRP-regulated splicing programme is abrogated during the epithelial-mesenchymal transition. Embo J.

18. Da Wei Huang B, Lempicki R (2008) Systematic and integrative analysis of large gene lists using DAVID bioinformatics resources.

19. Dennis Jr G, Sherman B, Hosack D, Yang J, Gao W, et al. (2003) DAVID: database for annotation, visualization, and integrated discovery. Genome Biol 4: P3.

20. Riaz M, Elstrodt F, Hollestelle A, Dehghan A, Klijn JG, et al. (2009) Low-risk susceptibility alleles in 40 human breast cancer cell lines. BMC Cancer 9: 236.

21. Blick T, Widodo E, Hugo H, Waltham M, Lenburg ME, et al. (2008) Epithelial mesenchymal transition traits in human breast cancer cell lines. Clin Exp Metastasis 25: 629-642.

22. de Hoon M, Imoto S, Nolan J, Miyano S (2004) Open source clustering software. Bioinformatics 20: 1453-1454.

23. Saldanha A (2004) Java Treeview--extensible visualization of microarray data. Bioinformatics 20: 3246.

24. de Hoon MJ, Imoto S, Nolan J, Miyano S (2004) Open source clustering software. Bioinformatics 20: 1453-1454.

25. Suzuki R, Shimodaira H (2006) Pvclust: an R package for assessing the uncertainty in hierarchical clustering. Bioinformatics 22: 1540-1542.

26. Warzecha CC, Shen S, Xing Y, Carstens RP (2009) The epithelial splicing factors ESRP1 and ESRP2 positively and negatively regulate diverse types of alternative splicing events. RNA Biol 6: 546-562.
